# Phosphotransferase-dependent accumulation of (p)ppGpp in response to glutamine deprivation in *Caulobacter crescentus*

**DOI:** 10.1038/ncomms11423

**Published:** 2016-04-25

**Authors:** Séverin Ronneau, Kenny Petit, Xavier De Bolle, Régis Hallez

**Affiliations:** 1 Bacterial Cell cycle and Development (BCcD), URBM, University of Namur, 61 Rue de Bruxelles, Namur 5000, Belgium

## Abstract

The alarmone (p)ppGpp is commonly used by bacteria to quickly respond to nutrient starvation. Although (p)ppGpp synthetases such as SpoT have been extensively studied, little is known about the molecular mechanisms stimulating alarmone synthesis upon starvation. Here, we describe an essential role of the nitrogen-related phosphotransferase system (PTS^Ntr^) in controlling (p)ppGpp accumulation in *Caulobacter crescentus*. We show that cells sense nitrogen starvation by way of detecting glutamine deprivation using the first enzyme (EI^Ntr^) of PTS^Ntr^. Decreasing intracellular glutamine concentration triggers phosphorylation of EI^Ntr^ and its downstream components HPr and EIIA^Ntr^. Once phosphorylated, both HPr∼P and EIIA^Ntr^∼P stimulate (p)ppGpp accumulation by modulating SpoT activities. This burst of second messenger primarily impacts the non-replicative phase of the cell cycle by extending the G1 phase. This work highlights a new role for bacterial PTS systems in stimulating (p)ppGpp accumulation in response to metabolic cues and in controlling cell cycle progression and cell growth.

## Abstract (web summary)

The small molecule (p)ppGpp is commonly produced by bacteria as a signal of nutrient starvation. Here, Ronneau *et al*. show that (p)ppGpp accumulation in the model bacterium *Caulobacter crescentus* is modulated by a nitrogen-related phosphotransferase system in response to glutamine deprivation.

## Graphical abstract

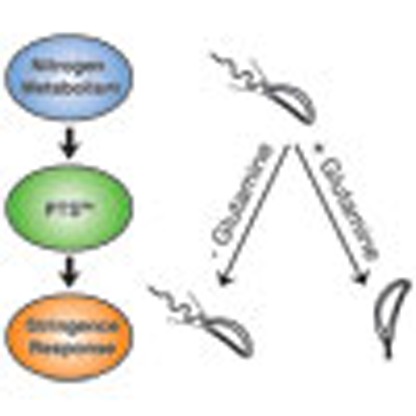

To face the environmental changes, organisms have developed complex regulatory mechanisms that integrate stimuli and stresses. Once activated, these signalling pathways modulate essential cellular processes such as DNA replication, cell division or cell growth. For example, upon nutrient starvation, yeast cells access to a specific quiescent state that enhances stress resistance and survival^1^. Bacteria also select many strategies to survive in challenging environments. One of the most studied bacterial adaptations to harsh conditions is certainly the formation of endospores in *Bacillus subtilis*, which requires asymmetric cell division and differentiation of the prespore^2^. Other bacteria take advantage of their asymmetric cell division to adapt to starvation conditions. It is the case of the aquatic α-proteobacterium *Caulobacter crescentus* that divides asymmetrically to give birth to two different daughter cells: a chemotactically active motile swarmer cell and a sessile stalked cell. Whereas the stalked cell grows and reinitiates DNA replication immediately at birth to ultimately divide again, the newborn swarmer cell enters first into a pre-replicative (G1) phase (Fig. 1a). In nutrient-replete conditions, the G1/swarmer cell differentiates into a stalked cell (swarmer-to-stalked transition) and concomitantly initiates chromosome replication (G1-to-S transition)^3^. Upon nitrogen starvation, *C. crescentus* extends its swarmer phase to promote spreading and colonization of new environments^4^,^5^,^6^. Asymmetric cell division might be a strategy commonly used by α-proteobacteria to generate daughter cells with different cell fates^7^.

Nutritional stresses are also known to be associated with the accumulation of an alarmone, the guanosine tetra- and penta-phosphate commonly called (p)ppGpp. Burst of intracellular (p)ppGpp alarmone allows cells to quickly adapt to a nutrient stress by affecting important cellular processes such as transcription, translation or DNA replication (reviewed in refs 8 and 9). For example, (p)ppGpp interferes with cell cycle steps by the direct binding of the alarmone to the DNA primase DnaG, which stops DNA replication in *B. subtilis*^10^. As a consequence of its pivotal role in stress adaptation, (p)ppGpp became crucial for virulence of several bacterial pathogens, long-term persistence, competence and biofilm formation^8^,^9^,^11^.

In *Escherichia coli*, (p)ppGpp level is regulated by two proteins, RelA and SpoT^12^. RelA is a monofunctional (p)ppGpp synthetase stimulated by amino acids starvation, in contrast to SpoT, which is a bifunctional synthetase-hydrolase enzyme responding to a wide range of nutritional stresses such as carbon, phosphate or fatty acid starvation^9^. *C. crescentus* possesses a single RelA/SpoT homologue^12^ that was named SpoT because of its bifunctional activity^13^,^14^. Previous studies showed that (p)ppGpp can modulate cell cycle progression in *C. crescentus* by delaying simultaneously the swarmer-to-stalked differentiation and the G1-to-S transition^13^,^15^,^16^. Nitrogen or carbon starvation was described to trigger accumulation of (p)ppGpp but the regulatory networks sensing these stresses and activating SpoT remain uncovered^13^,^14^. Furthermore, interacting partners of SpoT_Cc_ are largely unknown even if SpoT_Cc_ was shown to co-fractionate with the 70S ribosomal subunit^14^.

Ammonium (NH_4_^+^) is the preferred inorganic nitrogen source for most of living cells. There are only two reactions that efficiently assimilate NH_4_^+^ (Fig. 1b). The first one is catalysed by the NADP-dependent assimilative glutamate dehydrogenase. The other one is mediated by the ATP-dependent glutamine synthetase (GlnA). There is no NADP-dependent glutamate dehydrogenase encoded in the genome of *C. crescentus*, suggesting that the assimilation of inorganic nitrogen is strictly dependent on the glutamine synthetase (GlnA) activity. In most bacteria, nitrogen metabolism is tightly regulated by a well-characterized pathway, which involves the universal nitrogen sensor GlnD (Fig. 1b, reviewed in refs 17 and 18). When *E. coli* is grown in nitrogen-deplete (−N) conditions, the PII uridyltransferase GlnD catalyses the transfer of uridine monophosphate (UMP) groups to PII regulatory proteins, GlnB and GlnK. GlnK∼UMP no longer inhibits the ammonia channel AmtB, and GlnB∼UMP stimulates deadenylylation of GlnA by the adenylyltransferase GlnE, and thereby promotes glutamine synthetase activity (Fig. 1b). In nitrogen-replete (+N) conditions, GlnB inhibits the transcription of *glnA*, by stimulating dephosphorylation of the transcriptional activator NtrC, and promotes the addition of the adenine monophosphate groups by GlnE to GlnA, which slows down the glutamine synthetase activity (Fig. 1b).

In this work, we unravel the regulatory network that stimulates (p)ppGpp accumulation in *C. crescentus* in response to nitrogen starvation. In particular, we uncover the essential role of the nitrogen-related phosphoenolpyruvate (PEP) phosphotransferase system (PTS^Ntr^) in transducing glutamine deprivation signal to (p)ppGpp accumulation, which in turn controls the cell cycle progression. The cell cycle control described here constitutes a new PTS^Ntr^-dependent regulatory role, illustrating the diversity of the cellular processes regulated by PTS systems.

## Results

### Glutamine deprivation signals nitrogen starvation

Previous studies showed that nitrogen starvation extends the swarmer cell lifetime in *C. crescentus*^4^,^5^,^6^. By following DNA content and cell cycle-regulated proteins (the flagellin and the stalked-associated protein StpX) in synchronous or asynchronous population of *Caulobacter* cells, we confirmed the specific extension of the G1/swarmer phase in response to nitrogen starvation (Supplementary Fig. 1a–e). By contrast, stalked cells can complete DNA replication once initiated, despite the absence of a nitrogen source (Supplementary Fig. 1f).

To understand how nitrogen starvation affects the differentiation of G1/swarmer cells, we focused our work on proteins involved in nitrogen assimilation and metabolism. First, we created an in-frame deletion of the predicted gene coding for the general sensor for nitrogen availability, *glnD* (CCNA_00013). In contrast to wild-type cells, Δ*glnD* cells were unable to use ammonium as a nitrogen source. Indeed, the Δ*glnD* mutant did not grow and accumulated G1/swarmer cells when ammonium was used as the sole nitrogen source (Supplementary Fig. 2a,b). As expected, the G1 block and the growth were rescued in the presence of glutamine (Supplementary Fig. 2). Indeed, as for *glnD* mutants in *E. coli*^19^, *C. crescentus* Δ*glnD* is auxotrophic for glutamine. Thus this result shows that G1/swarmer cells accumulation in Δ*glnD* is a consequence of its inability to use ammonium as a nitrogen source. Interestingly, the Δ*glnD* mutant strain cultivated in a complex peptone yeast extract (PYE) medium accumulated G1/swarmer cells (Fig. 2c,d). As a consequence, Δ*glnD* cells also exhibited (i) a slower growth than the wild-type strain and (ii) a bigger motility halo than the wild-type, despite the growth defect (Fig. 2a,b). Indeed, the overrepresentation of the G1/swarmer cells in a Δ*glnD* population increases the overall motility and the doubling time of the strain. Thus, our results indicate that G1/swarmer lifetime is extended in the absence of *glnD* (Fig. 2a–d). Again, addition of glutamine suppressed all these phenotypes (Fig. 2d), suggesting that defects of Δ*glnD* are a consequence of glutamine availability in the complex PYE medium. Indeed, PYE is mainly composed of yeast extract, in which glutamine is the less-abundant amino acid (≤0.2%, see the ‘Methods' section).

**Figure 1:**
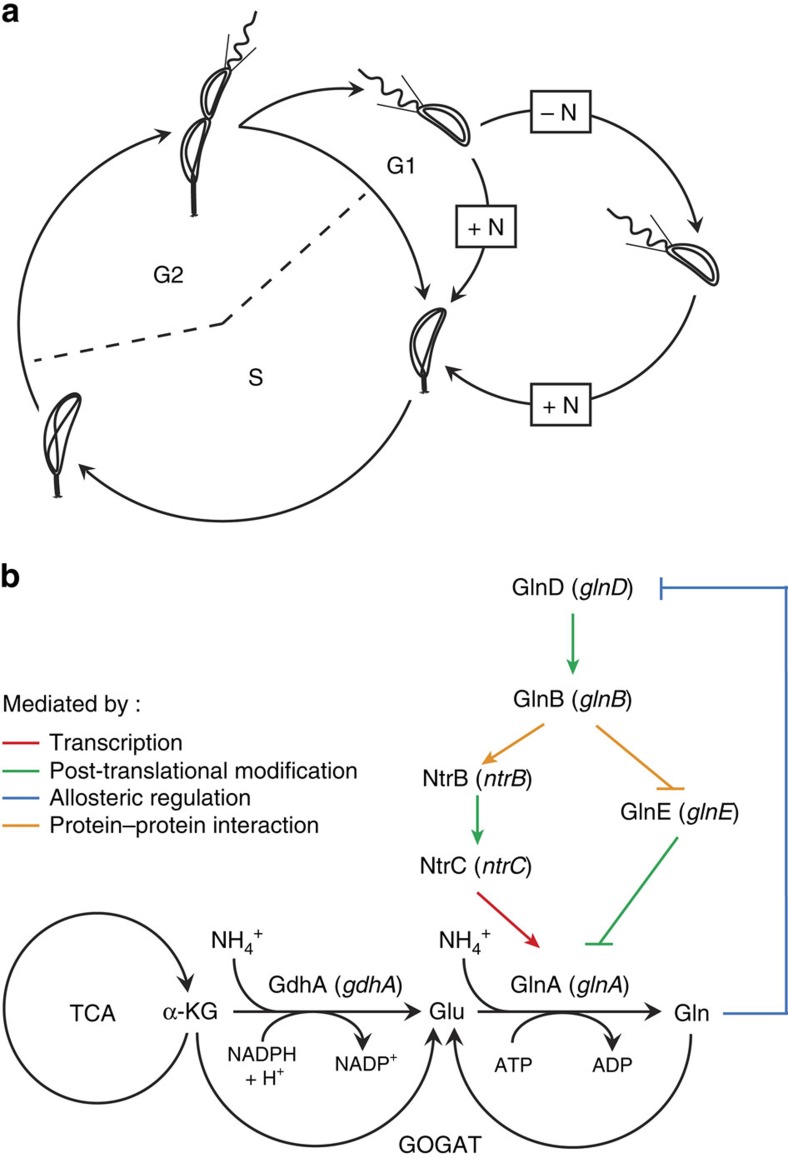
The *C. crescentus* swarmer cell lifetime is extended upon nitrogen starvation. (**a**) Asymmetric cell division of *C. crescentus* gives birth to a non-replicative swarmer cell that goes through G1 phase before replicating and a replicative stalked cell that directly enters into S phase. Upon nitrogen starvation (−N), swarmer cells extend their G1 phase. (**b**) In *E. coli*, ammonium can be assimilated either by the NADP-dependent glutamate dehydrogenase (GdhA) to generate glutamate (Glu) from α-ketoglutarate (α-KG) or by the glutamine synthetase (GlnA) to produce glutamine (Gln) from Glu, this latter being recycled by the glutamate synthase (GOGAT). GlnA is regulated at different levels by the GlnD/GlnB/GlnE and NtrBC pathways, and GlnD senses intracellular pool of Gln.

**Figure 2:**
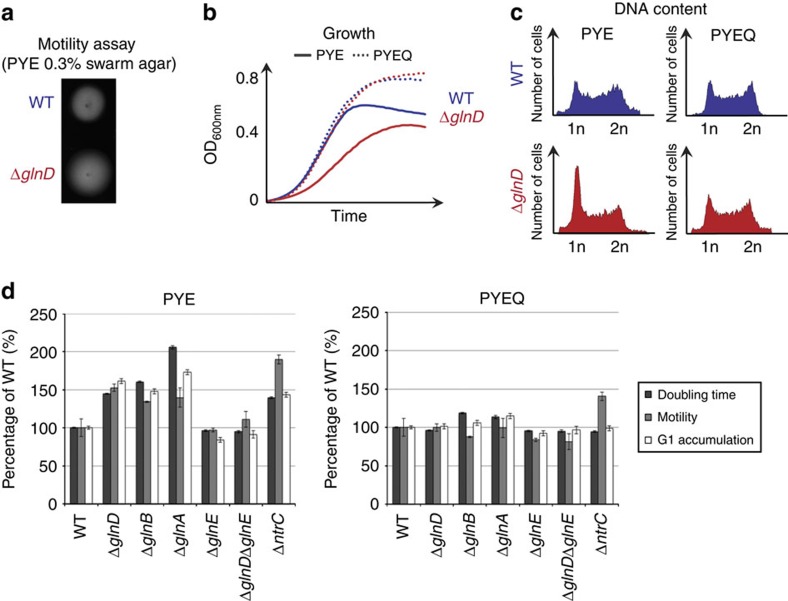
The *C. crescentus* G1/swarmer cell lifetime is dictated by intracellular glutamine concentration. (**a**–**d**) Extension of the G1/swarmer lifetime in a Δ*glnD* strain can be compensated by addition of glutamine. Motility (**a**), growth (**b**) and DNA content (**c**) of wild-type (WT; RH50) and Δ*glnD* (RH577) grown in complex media without (PYE) or with glutamine (PYEQ) media. (**d**) G1/swarmer lifetime is extended in glutamine auxotrophic mutants. Doubling time, motility and G1 proportion were measured in WT (RH50), Δ*glnD* (RH577), Δ*glnB* (RH771), Δ*glnA* (RH772), Δ*glnE* (RH874), Δ*glnD* Δ*glnE* (RH875) and Δ*ntrC* (RH1458) grown in complex media without (PYE) or with glutamine (PYEQ), and normalized to the WT (100%). All these phenotypes can be rescued by addition of glutamine (PYEQ). Error bars=s.d.; *n*=3.

In *E. coli*, glutamine auxotrophy displayed by *glnD* mutant strains comes from the under-expression and lower activity of the glutamine synthetase GlnA. In the absence of GlnD, the PII protein GlnB is not uridylylated, and thereby constitutively stimulates (i) the dephosphorylation of transcriptional activator NtrC∼P by NtrB, which subsequently inhibits the NtrC∼P-dependent expression of *glnA*, and (ii) the adenylylation (+adenine monophosphate) of GlnA by the adenylytransferase GlnE, thereby inhibiting the glutamine synthetase activity (Fig. 1b). *C. crescentus* encodes three PII protein homologues (*glnB* CCNA_02046, *glnK* CCNA_01400 and *glnC* CCNA_00555), one adenylytransferase homologue (*glnE* CCNA_02839), three GlnA homologues (*glnA* CCNA_02047, *glnA*_*2*_ CCNA_03230 and *glnA*_*3*_ CCNA_03240) and two NtrC homologues (*ntrC* CCNA_01815, and *ntrX* CCNA_01817). Single in-frame deletions of each of these genes were created and tested for growth, motility and G1 accumulation in complex PYE medium. We found that only Δ*glnB*, Δ*ntrC* and Δ*glnA* recapitulated Δ*glnD* phenotypes, and that all these phenotypes could be suppressed by adding glutamine to the medium (Fig. 2d). However, it is noteworthy that the motility defect in Δ*ntrC* was not completely rescued by addition of glutamine, suggesting that NtrC also controls motility independently of G1/swarmer cells accumulation (Fig. 2d). Moreover, deleting *glnE* alleviated the Δ*glnD* defects, supporting the fact that inactivation (adenylylation) of GlnA by GlnE is the causative effect of the glutamine auxotrophy detected in Δ*glnD* cells (Fig. 2d). Unexpectedly, neither GlnA_2_ nor GlnA_3_ seems to participate in glutamine synthesis, at least in the conditions used here (Supplementary Fig. 3a,b). Finally, a catalytic mutant inactivating the glutamine synthetase activity of GlnA (GlnA_R360A_) phenocopied Δ*glnA* in terms of glutamine auxotrophy, growth defect and G1 accumulation (Supplementary Fig. 3c,d), and all these defects can be suppressed by the addition of glutamine or complemented with a wild-type copy of *glnA* expressed *in trans* (Supplementary Fig. 3d,e). Altogether, these data support that (i) glutamine deprivation constitutes the signal for nitrogen starvation and that (ii) intracellular levels of glutamine drive the cell cycle progression of *C. crescentus*.

### Cell cycle response to nitrogen starvation requires (p)ppGpp

**Figure 3:**
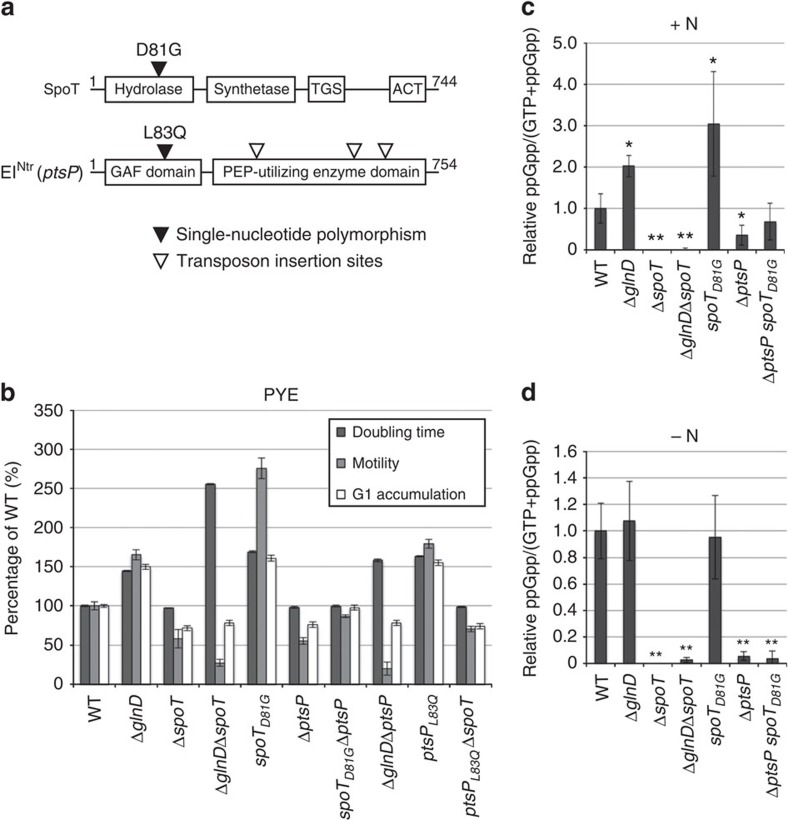
SpoT and EI^Ntr^ modulate (p)ppGpp accumulation on nitrogen starvation. (**a**) Mutations isolated in SpoT and EI^Ntr^ that modulate G1/swarmer lifetime. Black arrowheads indicate the mutations (*spoT*_*D81G*_ and *ptsP*_L83Q_) that increase G1/swarmer lifetime. White arrowheads indicate the positions of 3 *himar* transposon insertions in the *ptsP* locus out of the 16 that suppressed *spoT*_*D81G*_ phenotypes. The exact positions of the remaining 13 transposon insertions into the *ptsP* locus were not determined. (**b**) SpoT and EI^Ntr^ control G1/swarmer lifetime. Doubling time, motility and G1 proportion were measured in wild-type (WT; RH50), Δ*glnD* (RH577), Δ*spoT* (RH1755), Δ*glnD* Δ*spoT* (RH1756), *spoT*_*D81G*_ (RH1752), Δ*ptsP* (RH1758), Δ*ptsP spoT*_*D81G*_ (RH1727) Δ*glnD* Δ*ptsP* (RH1940), *ptsP*_L83Q_ (RH1748) and Δ*spoT ptsP*_L83Q_ (RH1728) grown in complex media (PYE) and normalized to the WT (100%). Error bars=s.d.; *n*=3. (**c**,**d**) Glutamine auxotrophy leads to (p)ppGpp accumulation. Intracellular levels of (p)ppGpp detected by TLC after nucleotides extraction of WT (RH50), Δ*glnD* (RH577), Δ*spoT* (RH1755), Δ*glnD* Δ*spoT* (RH1756), *spoT*_*D81G*_ (RH1752), Δ*ptsP* (RH1758) and Δ*ptsP spoT*_*D81G*_ (RH1727) grown (**c**) in +N or (**d**) −N conditions. Error bars=s.d.; *n*=3. Statistically significant differences by Student's *t*-test in comparison with the WT are indicated as *P<0.05% and **P<0.01% (*n*=3).

To fish out key actors participating to the G1/swarmer extension in response to glutamine deprivation, we isolated spontaneous mutations that increase motility of the wild-type strain on PYE swarm agar plates supplemented with glutamine. Whole-genome sequencing of one candidate revealed a unique mutation (D81G) in the hydrolase domain of SpoT, the protein synthesizing (p)ppGpp (Fig. 3a and Supplementary Fig. 4a). It has been shown that mutations abolishing, at least partially, the hydrolase activity of SpoT without altering its synthetase activity, were all found in the hydrolase domain^20^. Interestingly, the conserved aspartate, corresponding to the D81 in *C. crescentus* SpoT, was found to be required for the hydrolase activity of SpoT in *Streptococcus dysgalactiae*^20^, which suggests that the D81G mutation may reduce hydrolase activity of SpoT in *C. crescentus* as well. The *spoT*_*D81G*_ strain had a growth delay in PYE and accumulated G1/swarmer cells in exponential phase of growth (Fig. 3b; Supplementary Fig. 5a–c and Supplementary Movie 1), even when glutamine was added to the medium (Supplementary Figs 5a–c and 6), confirming that the *spoT*_*D81G*_ mutant is insensitive to glutamine. By contrast, in-frame deletion of *spoT* (Δ*spoT*) displayed a motility defect on swarm agar and contained less G1/swarmer cells, in comparison with the wild-type (Fig. 3b; Supplementary Fig. 5b,c and Supplementary Movie 1). Most importantly, introducing Δ*spoT* in Δ*glnD* cells completely suppressed the accumulation of G1/swarmer cells (Fig. 3b and Supplementary Fig. 5b,c). This result highlights the role of (p)ppGpp produced by SpoT in response to glutamine deprivation to control the cell cycle. We thus checked the (p)ppGpp production in −N or +N conditions. In agreement with previous studies^14^, we found that (p)ppGpp concentration increases on glutamine deprivation, that is, in the wild-type strain grown without nitrogen source (−N) or in Δ*glnD* cultivated with (+N) or without (−N) NH_4_^+^ (Fig. 3c,d; Supplementary Figs 1g and 5d). As already mentioned in previous works^13^,^15^, we also observed a low amount of (p)ppGpp produced by wild-type cells in non-stressed conditions (Fig. 3c and Supplementary Fig. 5d). This (p)ppGpp steady-state level was slightly higher in the *spoT*_*D81G*_ strain in +N conditions (Fig. 3c and Supplementary Fig. 5d), which would explain the phenotypes displayed by *spoT*_*D81G*_, that is, slower growth, bigger motility halo and G1 accumulation (Fig. 3b), even in the presence of glutamine (Supplementary Figs 5a–c and 6). Nevertheless, *spoT*_*D81G*_ cells accumulated similar levels of (p)ppGpp than wild-type cells upon nitrogen starvation (−N), suggesting that SpoT_D81G_ is still sensitive to nitrogen starvation (Fig. 3d). In contrast, the disappearance of (p)ppGpp accumulated upon nitrogen starvation is slower in *spoT*_*D81G*_ cells than in wild-type cells (Supplementary Fig. 5e). These results support that D81G mutation mainly affects the hydrolase activity of SpoT. It's noteworthy that an artificial increase of (p)ppGpp levels in non-starved cells displayed similar phenotypes than *spoT*_*D81G*_ in complex medium PYE^16^. The fact that, in non-stressed conditions, a strain producing more (p)ppGpp (*spoT*_*D81G*_) accumulated G1/swarmer cells, whereas a strain producing no (p)ppGpp at all (Δ*spoT*) contained less G1/swarmer cells (Fig. 3b,c), suggest that (p)ppGpp steady-state level might determine the time spent by swarmer cells in G1 phase. Altogether, our findings indicate that glutamine deprivation increases (p)ppGpp level, which in turn, will extend the lifetime of G1/swarmer cells.

### PTS^Ntr^ promotes (p)ppGpp accumulation on nitrogen starvation

To identify factors that participate to the activation of SpoT in response to nitrogen starvation, we selected for transposon insertions that improve growth of *spoT*_*D81G*_ cells on complex medium (PYE). Indeed, the accumulation of (p)ppGpp in *spoT*_*D81G*_ cells decreases the growth rate on PYE medium (Fig. 3b and Supplementary Fig. 5a). We identified multiple transposon insertions (34 out of 50 clones) into *spoT*_*D81G*_ itself. The remaining 16 clones harboured a transposon insertion into the *ptsP* gene (*CCNA_00892*), coding for a nitrogen-related PEP-phosphotransferase (PTS) protein homologue, called PtsP or EI^Ntr^ in Enterobacteria (Fig. 3a). Canonical PTS systems are composed of several components that form a phosphorylation cascade initiated by autophosphorylation of the first protein called EI, using PEP as phosphoryl donor (reviewed in ref. 21). The phosphoryl group is then transferred from EI∼P to HPr and then to EIIA proteins. When the PTS system is used to take up sugars, the phosphoryl group is ultimately transferred from EIIA∼P to transported carbohydrates by using specific permeases (EIIB and EIIC components). In many other cases, PTS systems are dedicated to regulatory functions implying that PTS components (EI, HPr or EIIA) phosphorylate or interact with regulatory target proteins^21^. Nitrogen-related PTS (PTS^Ntr^) systems are so far considered as unusual PTS systems that respond to nitrogen availability, but their regulatory roles in bacterial physiology remain poorly understood (reviewed in ref. 21).

An in-frame deletion of *ptsP* in the parental *spoT*_*D81G*_ strain suppressed the *spoT*_*D81G*_ phenotypes, confirming the genetic interaction between *ptsP* and *spoT*_*D81G*_ (Fig. 3b). In addition, Δ*ptsP* phenocopied Δ*spoT* in terms of motility, G1/swarmer cells accumulation (Fig. 3b; Supplementary Figs 5a–c and 7a–c), and capability to suppress G1/swarmers cells accumulation of Δ*glnD* cells (compare Δ*glnD* Δ*ptsP* to Δ*glnD* Δ*spoT* in Fig. 3b; Supplementary Figs 5a–c and 7a–c). Interestingly, we isolated another candidate than *spoT*_*D81G*_ in the gain-of-motility screen, which harboured a mutation (L83Q) in the GAF domain of EI^Ntr^ (*ptsP*_*L83Q*_; Fig. 3a). As *ptsP*_*L83Q*_ phenocopies *spoT*_*D81G*_ (Fig. 3b; Supplementary Figs 5a–c and 7a–c), Δ*ptsP* suppresses *spoT*_*D81G*_ defects and Δ*spoT* suppresses *ptsP*_*L83Q*_ defects (Fig. 3b), we wondered whether *ptsP* (EI^Ntr^) is upstream or downstream of *spoT*. To test that, we measured the (p)ppGpp levels in a *spoT*_*D81G*_ Δ*ptsP* background. As shown in Fig. 3d, no (p)ppGpp accumulation was detected in *spoT*_*D81G*_ Δ*ptsP* cells starved for nitrogen (−N). However, *spoT*_*D81G*_ Δ*ptsP* cells still produced a low amount of (p)ppGpp, whether a nitrogen source was added to the medium or not (Fig. 3c). This constitutive low levels of (p)ppGpp produced by *spoT*_*D81G*_ Δ*ptsP* cells is very close to the (p)ppGpp level detected in non-starved wild-type cells (Fig. 3c). Interestingly, there is a systematic correlation between the amount of (p)ppGpp produced by the cells and the time spent by these cells in G1 phase. Indeed, Δ*spoT* or Δ*ptsP* swarmer cells do not produce detectable levels of (p)ppGpp (Fig. 3c) and have shortened G1 phase (Fig. 3b), whereas *spoT*_*D81G*_ Δ*ptsP* and wild-type cells have similar levels of (p)ppGpp and G1 lifetime (Fig. 3b,c). Altogether, these results support the role played by (p)ppGpp in determining the G1 lifetime, and show that EI^Ntr^ regulates (p)ppGpp levels by controlling SpoT.

### Glutamine inhibits EI^Ntr^ autophosphorylation

**Figure 4:**
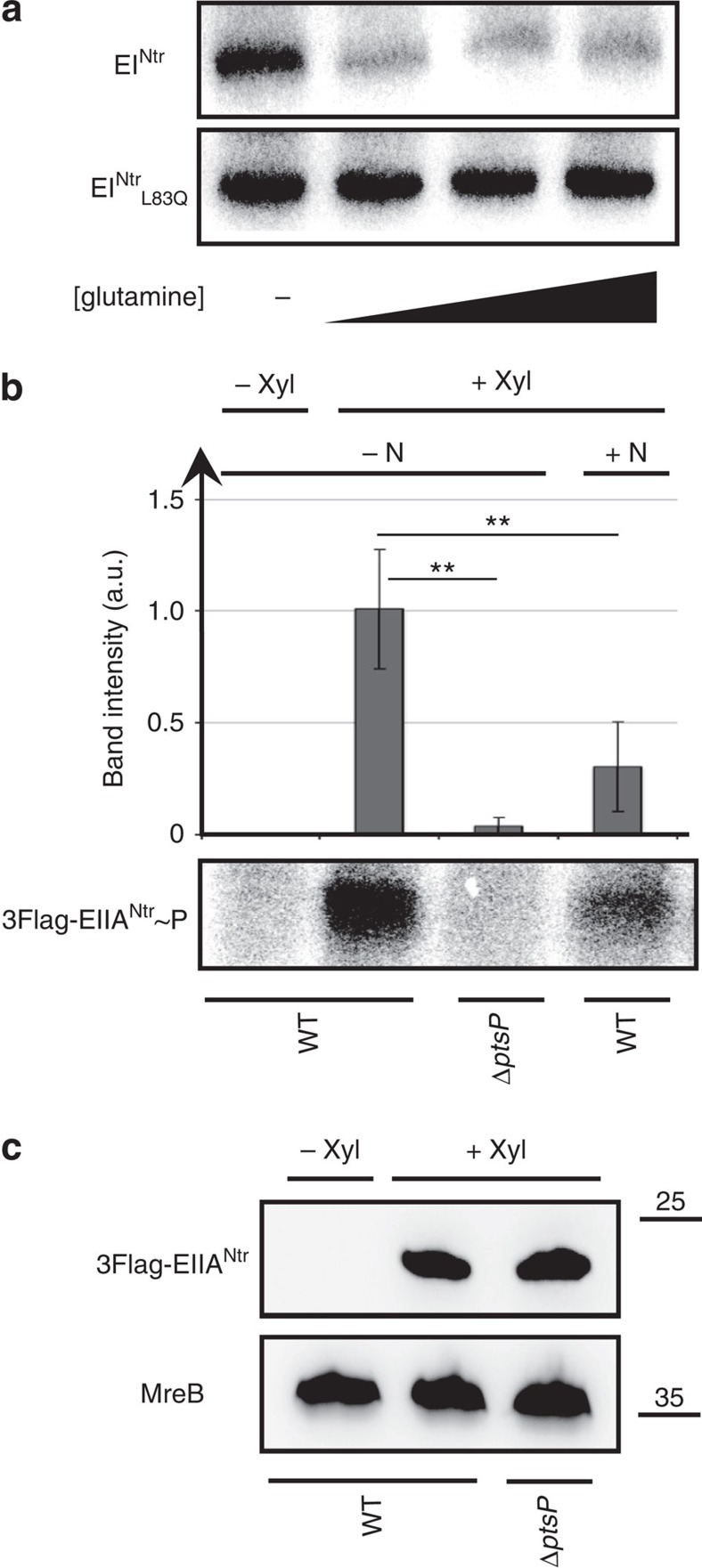
Glutamine inhibits PTS^Ntr^ phosphorylation. (**a**) Glutamine inhibits autophosphorylation of EI^Ntr^ but not EI^Ntr^_L83Q_. Autophosphorylation assays of EI^Ntr^ and EI^Ntr^_L83Q_ using [^32^P]PEP as a phosphoryl donor in the absence or presence of increasing concentration of glutamine (0, 2, 5, 10 mM). The full autoradiography is available in Supplementary Fig. 8a. (**b**) The EI^Ntr^-dependent phosphorylation of EIIA^Ntr^ is enhanced upon nitrogen starvation. *In vivo* phosphorylation assays of EIIA^Ntr^ in −N or +N conditions supplemented with (+Xyl) or without (−Xyl) xylose in wild-type (WT) or Δ*ptsP* cells expressing *3FLAG-ptsN* from P*xylX* promoter. Error bars=s.d.; *n*=3. Statistically significant differences by Student's *t*-test are indicated as **P<0.01% (*n*=3). The full autoradiography is available in Supplementary Fig. 8b. (**c**) Immunoblotting of protein samples extracted from WT and Δ*ptsP* cells expressing *3FLAG-ptsN* from P*xylX* promoter, incubated 3 h in M5GG supplemented with (+Xyl) or without (−Xyl) xylose. MreB was detected in all conditions, while 3FLAG-EIIA^Ntr^ was detected only in the presence of xylose. The full blot is available in Supplementary Fig. 8c.

To understand how glutamine deprivation is transduced to SpoT, we first looked at the autophosphorylation level of EI^Ntr^. Indeed, as described above, accumulation of G1/swarmer cells (Fig. 3b) observed in a *ptsP*_*L83Q*_ background, are not compensated by supplying an exogenous source of glutamine (Supplementary Fig. 6). Moreover, it has been recently shown, in the closely related α-proteobacterium *Sinorhizobium meliloti*, that binding of glutamine to the conserved N-terminal GAF domain of EI^Ntr^ inhibits its autophosphorylation^22^. To check whether the phosphorylation of EI^Ntr^ is also sensitive to glutamine in *C. crescentus*, we performed *in vitro* autophosphorylation assays with a purified fraction containing EI^Ntr^ using [^32^P]PEP as a phosphoryl donor, in the presence or absence of glutamine. We found that autophosphorylation of EI^Ntr^ was strongly reduced by glutamine (Fig. 4a and Supplementary Fig. 8a). In contrast, autophosphorylation of the EI^Ntr^_L83Q_ mutant form was not modulated by the presence of glutamine (Fig. 4a), suggesting that the mutation L83Q prevents glutamine binding to the highly conserved region of the EI^Ntr^ GAF domain (Supplementary Fig. 4b).

### Phosphorylated PTS^Ntr^ proteins trigger (p)ppGpp accumulation

**Figure 5:**
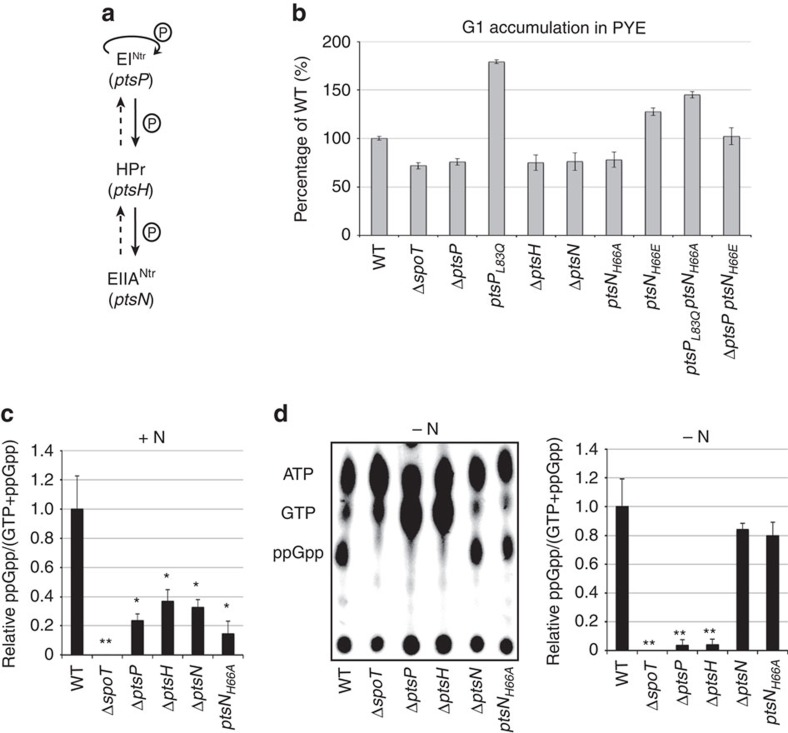
PTS^Ntr^ modulates (p)ppGpp accumulation. (**a**) Schematic representation of the PTS^Ntr^ pathway in *C. crescentus*. (**b**) The PTS^Ntr^ components EI^Ntr^, HPr and EIIA^Ntr^ control G1/swarmer lifetime. G1 proportion was measured in wild-type (WT; RH50), Δ*spoT* (RH1755), Δ*ptsP* (RH1758), *ptsP*_*L83Q*_ (RH1748), Δ*ptsH* (RH1621), Δ*ptsN* (RH1819), *ptsN*_*H66A*_ (RH2019), *ptsN*_*H66E*_ (RH2017), *ptsP*_*L83Q*_ *ptsN*_*H66A*_ (RH2018), Δ*ptsP ptsN*_*H66E*_ (RH2016) grown in complex media (PYE) and normalized to the WT (100%). Error bars=s.d.; *n*=3. (**c**,**d**) (p)ppGpp accumulation is modulated by PTS^Ntr^. Intracellular levels of (p)ppGpp detected by TLC after nucleotides extraction of WT (RH50), Δ*spoT* (RH1755), Δ*ptsP* (RH1758), Δ*ptsH* (RH1621), Δ*ptsN* (RH1819) and *ptsN*_*H66A*_ (RH2019) grown (**c**) in +N or (**d**) −N conditions. Error bars=s.d.; *n*=3. Statistically significant differences by Student's *t*-test in comparison with the WT are indicated as *P<0.05% and **P<0.01% (*n*=3).

To unravel how EI^Ntr^ controls SpoT activity, we first searched for components that could participate to PTS^Ntr^ phosphorelay (Fig. 5a). Besides *ptsP* (EI^Ntr^), we found a unique HPr homologue (*ptsH*, *CCNA_00241*) and another nitrogen-related PTS^Ntr^ component, EIIA^Ntr^ (*ptsN*, *CCNA_03710*). We created single in-frame deletions of the two genes (Δ*ptsH* and Δ*ptsN*) and found that the proportion of G1/swarmer cells in Δ*ptsH* (without HPr) or Δ*ptsN* (without EIIA^Ntr^) strain was reduced in comparison with the wild-type strain in complex medium (Fig. 5b), a phenotype already described for Δ*ptsP* (without EI^Ntr^) and for Δ*spoT* (Fig. 5b). Interestingly, strains expressing a non-phosphorylatable version of EIIA^Ntr^ (EIIA^Ntr^_H66A_) accumulated G1 cells as much as the loss-of-function mutant (Δ*ptsN*), that is, less than the wild-type strain (Fig. 5b), suggesting that the PTS^Ntr^ pathway is slightly phosphorylated in complex medium PYE and that the last component of the PTS^Ntr^ phosphorelay, EIIA^Ntr^, controls SpoT activity.

To validate the conservation of PTS^Ntr^ phosphorelay and the inhibitory effect of glutamine on this cascade, we checked the phosphorylation level of EIIA^Ntr^ in +N or −N conditions. To this purpose, we performed *in vivo* phosphorylation assays on WT and Δ*ptsP* (without EI^Ntr^) strains expressing a xylose-inducible tagged version of EIIA^Ntr^ (P*xylX::3FLAG-ptsN*; Fig. 4b,c and Supplementary Fig. 8b,c). In agreement with our previous data, we found that the phosphorylation of EIIA^Ntr^ is enhanced in the absence of nitrogen sources (−N) in comparison with the +N conditions (Fig. 4b,c and Supplementary Fig. 8b,c). In addition, we showed that EI^Ntr^ is required *in vivo* for EIIA^Ntr^ phosphorylation, since phosphorylated EIIA^Ntr^ was undetectable in Δ*ptsP* cells starved for nitrogen (Fig. 4b,c and Supplementary Fig. 8b,c).

In addition, we measured the (p)ppGpp levels in the single PTS^Ntr^ mutants first in +N conditions. Consistent with the G1 accumulation in PYE, we found that PTS^Ntr^ mutant strains (Δ*ptsP*, Δ*ptsH*, Δ*ptsN* or *ptsN*_*H66A*_) produced significantly lower amount of (p)ppGpp than the wild-type strain in the presence of a nitrogen source (Fig. 5c). Moreover, EIIA^Ntr^_H66A_ partially abrogated the cell cycle and developmental defects of *ptsP*_*L83Q*_ (EI^Ntr^_L83Q_) supporting the fact that overphosphorylation of EIIA^Ntr^ in *ptsP*_*L83Q*_ cells is partially responsible for (p)ppGpp accumulation and subsequent G1-to-S transition delay (Fig. 5b). On the contrary, a strain expressing a phosphomimetic mutant of EIIA^Ntr^ (EIIA^Ntr^_H66E_) had increased proportion of G1 cells independently of the presence of EI^Ntr^ (Fig. 5b). Altogether, these data suggest that the phosphorylated form of EIIA^Ntr^ (EIIA^Ntr^∼P) controls SpoT activity.

However, (p)ppGpp measurements in −N conditions showed that SpoT is also controlled in an EIIA^Ntr^∼P-independent way. Indeed, in contrast to cells devoid of EI^Ntr^ (Δ*ptsP*) or HPr (Δ*ptsH*), which did not accumulate (p)ppGpp upon nitrogen starvation (Fig. 5d), the absence of EIIA^Ntr^∼P in Δ*ptsN* or *ptsN*_*H66A*_ cells did not abolish (p)ppGpp accumulation upon nitrogen starvation (Fig. 5d), showing that SpoT is still sensitive to nitrogen availability in the absence of EIIA^Ntr^∼P. On the basis of these results, we propose a model in which HPr∼P controls the intracellular levels of (p)ppGpp by at least two ways, in an EIIA^Ntr^∼P-dependent way but also independently of EIIA^Ntr^∼P.

### Phosphorylated EIIA^Ntr^ directly interacts with SpoT

**Figure 6:**
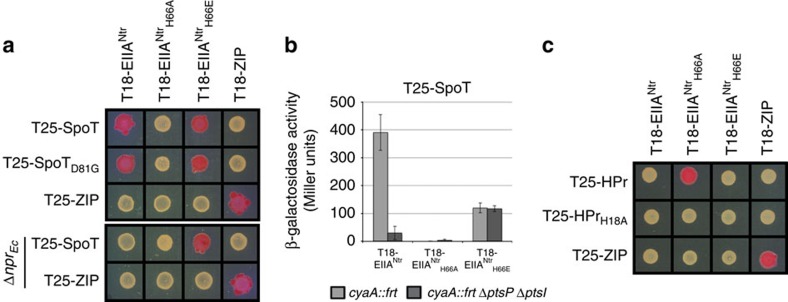
The phosphorylated form of EIIA^Ntr^ interacts with SpoT in BTH assays. (**a**) In contrast to EIIA^Ntr^_H66A_, EIIA^Ntr^∼P and EIIA^Ntr^_H66E_ directly interact with SpoT. MG1655 *cyaA::frt* (RH785) or MG1655 *cyaA::frt* Δ*npr* (RH2122) strains coexpressing T18- fused to *ptsN*, *ptsN*_*H66A*_*, ptsN*_*H66E*_ or *ZIP* with T25- fused to *spoT*, *spoT*_*D81G*_ or *ZIP* were spotted on MacConkey agar base plates supplemented with 1% maltose and 1 mM IPTG. Plates were incubated for 3–4 days at 30 °C. The red colour indicates positive interactions. (**b**) The absence of *E. coli* EI and EI^Ntr^ proteins abolishes interaction between SpoT and EIIA^Ntr^ but not with EIIA^Ntr^_H66E_. β-galactosidase assays were performed on strains coexpressing T25-SpoT and T18-EIIA^Ntr^, T18-EIIA^Ntr^_H66A_ or T18-EIIA^Ntr^_H66E_, in the presence (MG1655 *cyaA::frt* (RH785)) or absence (MG1655 *cyaA::frt* Δ*ptsP* Δ*ptsI* (RH2124)) of EI and EI^Ntr^ proteins. Error bars=s.d.; *n*=3. (**c**) In contrast to HPr_H18A_, HPr∼P directly interacts with EIIA^Ntr^_H66A_. MG1655 *cyaA::frt* (RH785) strain coexpressing T18- fused to *ptsN*, *ptsN*_*H66A*_*, ptsN*_*H66E*_ or *ZIP* with T25- fused to *hpr*, *hpr*_*H18A*_ or *ZIP* were spotted on MacConkey agar base plates supplemented with 1% maltose and 1 mM IPTG. Plates were incubated for 3–4 days at 30 °C. The red colour indicates positive interactions.

Since most of the regulatory functions of PTS components are mediated by protein–protein interactions, we checked whether HPr and EIIA^Ntr^ were able to interact with SpoT by performing bacterial two-hybrid (BTH) assays. To this end, T18 or T25 domains of *Bordetella pertussis* adenylate cyclase^23^ were fused to coding sequences of HPr (*ptsH* and *ptsH*_*H18A*_), EIIA^Ntr^ (*ptsN*, *ptsN*_*H66A*_ and *ptsN*_*H66E*_) and SpoT (*spoT* and *spoT*_*D81G*_). We found that both the wild-type EIIA^Ntr^ (*ptsN*) and the phosphomimetic mutant of EIIA^Ntr^ (EIIA^Ntr^_H66E_) were able to interact with SpoT versions (Fig. 6a,b and Supplementary Fig. 9a), while the non-phosphorylatable mutant EIIA^Ntr^_H66A_ was not (Fig. 6a,b and Supplementary Fig. 9a). Both T18-EIIA^Ntr^_H66A_ and T25-EIIA^Ntr^_H66A_ can, respectively, interact with T25-HPr and T18-HPr (Fig. 6c and Supplementary Fig. 9a), showing that EIIA^Ntr^_H66A_ is functional in the BTH assays. Altogether, these findings suggest that EIIA^Ntr^ is phosphorylated *in vivo* in *E. coli*. Indeed, there are two PTS systems in *E. coli*, a canonical one composed of EI (*ptsI*), HPr (*ptsH*) and EIIA (*ptsM*), as well as a nitrogen-related one composed of EI^Ntr^ (*ptsP*), NPr (*npr*) and EIIA^Ntr^ (*ptsN*), and both pathways can cross-talk to some extent^24^ (Supplementary Fig. 9b). To test whether *Caulobacter* EIIA^Ntr^ is phosphorylated *in vivo* in *E. coli*, strains deleted for *npr* (NPr) or for both *ptsP* (EI^Ntr^) and *ptsI* (EI) genes were created. As illustrated on Fig. 6a and Supplementary Fig. 9c, the interaction between EIIA^Ntr^ and SpoT was abolished in the Δ*npr* strain while EIIA^Ntr^_H66E_ retained the ability to interact with SpoT. Likewise, no more β-galactosidase activity was detected in a Δ*ptsP* Δ*ptsI* background (without *E. coli* EI proteins) expressing T25-SpoT and T18-EIIA^Ntr^, while the interaction between SpoT and EIIA^Ntr^_H66E_ remained unchanged in this background (Fig. 6b). Finally, the expression of *Caulobacter ptsH* (HPr) from the inducible pBAD promoter (pBAD33*-ptsH*_*Cc*_) in a Δ*npr* background restored the interaction between EIIA^Ntr^ and SpoT only when arabinose was added to the medium (Supplementary Fig. 9c), indicating that *Caulobacter* HPr and EIIA^Ntr^ proteins can be phosphorylated by *E. coli* PTS systems, and that only the phosphorylated form of EIIA^Ntr^ interacts with SpoT. In contrast to EIIA^Ntr^, no interaction was detected between HPr (or HPr_H18A_) and SpoT (or SpoT_D81G_) on MacConkey maltose agar plates (Supplementary Fig. 9d). The fact that HPr interacts with EIIA^Ntr^_H66A_ (Fig. 6c and Supplementary Fig. 9a) shows that HPr is functional in the BTH assays.

Altogether, these BTH data strongly suggest that (i) HPr and EIIA^Ntr^ are both phosphorylated in *E. coli* and (ii) EIIA^Ntr^∼P is the only form of EIIA^Ntr^ able to interact with SpoT, thereby supporting a model in which SpoT activity is controlled directly by EIIA^Ntr^∼P, and indirectly by HPr∼P (Fig. 8).

### Phosphorylated EIIA^Ntr^ inhibits hydrolase activity of SpoT

**Figure 7:**
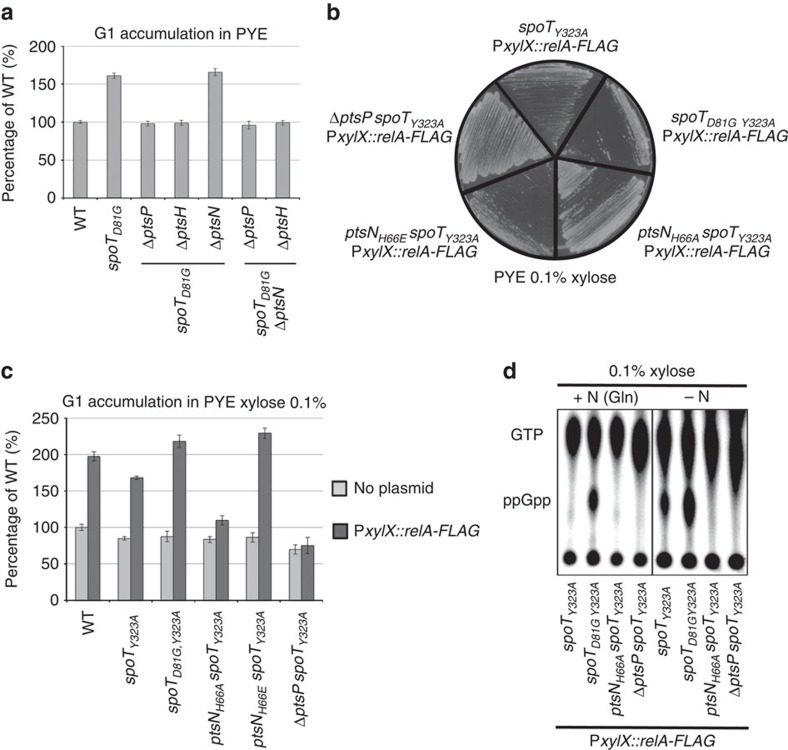
EIIA^Ntr^∼P inhibits the hydrolase activity of SpoT. (**a**) *spoT*_*D81G*_ is insensitive to the absence of EIIA^Ntr^. G1 proportion was measured in wild-type (WT; RH50), *spoT*_*D81G*_ (RH1752), *spoT*_*D81G*_ Δ*ptsP* (RH1727), *spoT*_*D81G*_ Δ*ptsH* (RH2013), *spoT*_*D81G*_ Δ*ptsN* (RH1999), *spoT*_*D81G*_ Δ*ptsN* Δ*ptsP* (RH2014) and *spoT*_*D81G*_ Δ*ptsN* Δ*ptsH* (RH2015) grown in complex media (PYE) and normalized to the WT (100%). Error bars=s.d.; *n*=3. (**b**) The hydrolase activity of SpoT is required for growth on an artificial exogenous production of (p)ppGpp. Growth of *spoT*_*Y323A*_*, spoT*_*D81G Y323A*_*, ptsN*_*H66A*_*spoT*_*Y323A*_*, ptsN*_*H66E*_*spoT*_*Y323A*_ and Δ*ptsP spoT*_*Y323A*_ expressing a truncated version of *E. coli relA* from the inducible *xylX* promoter (P*xylX::relA-FLAG*) on PYE medium supplemented with 0.1% of xylose. (**c**) Reduction of SpoT hydrolase activity led to a G1 extension on an artificial exogenous production of (p)ppGpp. Flow cytometry analysis to determine DNA content in asynchronous population of WT, *spoT*_*Y323A*_*, spoT*_*D81G Y323A*_*, ptsN*_*H66A*_*spoT*_*Y323A*_*, ptsN*_*H66E*_*spoT*_*Y323A*_ and Δ*ptsP spoT*_*Y323A*_ with (dark grey bars) or without (light grey bars) P*xylX::relA-FLAG* in PYE medium supplemented with 0.1% of xylose. The data were normalized to the WT without P*xylX::relA-FLAG* (100%). Error bars=s.d.; *n*=3. (**d**) The hydrolase activity of SpoT is required to degrade (p)ppGpp in +N condition. Intracellular levels of (p)ppGpp detected by TLC after nucleotides extraction of *spoT*_*Y323A*_*, spoT*_*D81G Y323A*_*, ptsN*_*H66A*_*spoT*_*Y323A*_ and Δ*ptsP spoT*_*Y323A*_ containing P*xylX::relA-FLAG* in +N or −N medium supplemented with 0.1% xylose.

Interestingly, the deletion of *ptsN* (EIIA^Ntr^) did not abolish the G1 accumulation of *spoT*_*D81G*_ cells in contrast to Δ*ptsP* (EI^Ntr^) or Δ*ptsH* (HPr; Fig. 7a). The fact that SpoT_D81G_, which harbours a reduced hydrolase activity (Supplementary Fig. 5e), is insensitive to the presence of EIIA^Ntr^ suggests that EIIA^Ntr^∼P might inhibit the hydrolase activity of SpoT rather than stimulating its synthetase activity. This could explain why Δ*ptsN* or *ptsN*_*H66A*_ are still able to accumulate high levels of (p)ppGpp on nitrogen starvation, while Δ*ptsP* or Δ*ptsH* cannot (Fig. 5d). To validate our hypothesis, we engineered *Caulobacter* strains in which the only (p)ppGpp synthetase activity was supplied by the unrelated *E. coli* RelA protein, and we measured the endogenous hydrolase activity of SpoT in different genetic backgrounds. To this end, we first abolished the synthetase activity of SpoT in several backgrounds (*spoT*_*D81G*_, *ptsN*_*H66A*_, *ptsN*_*H66E*_ and Δ*ptsP*), by replacing the tyrosine 323 of SpoT by an alanine (SpoT_Y323A_; ref. 14). As expected, all these strains displayed a G1 accumulation similar to a Δ*spoT* strain in PYE complex medium (Fig. 7c). In a second time, we inserted a truncated version of *E. coli* RelA (p)ppGpp synthetase at the xylose locus, leading to an artificial (p)ppGpp accumulation in *Caulobacter* upon addition of xylose (P*xylX::relA-FLAG*; ref. 16). Since the hydrolase domain of RelA is inactive, the only (p)ppGpp hydrolase activity in these strains was carried out by the *Caulobacter* SpoT protein, while the only (p)ppGpp synthetase activity was supported by the *E. coli* RelA protein. In the presence of xylose, both *spoT*_*D81G Y323A*_ and *ptsN*_*H66E*_ *spoT*_*Y323A*_ displayed a growth defect and a G1 accumulation in comparison with the parental *spoT*_*Y323A*_ strain (Fig. 7b,c and Supplementary Fig. 10). On the contrary, neither *ptsN*_*H66A*_ *spoT*_*Y323A*_ nor Δ*ptsP spoT*_*Y323A*_ strains had a growth delay or accumulated G1/swarmer cell upon xylose induction. These results strongly suggest that the phosphorylated form of EIIA^Ntr^ (*ptsN*) specifically inhibits the hydrolase activity of SpoT. In support of this, we found that upon xylose induction (Fig. 7d), *spoT*_*Y323A*_ P*xylX::relA-FLAG* cells accumulated (p)ppGpp in −N conditions (that is, when EIIA^Ntr^ is highly phosphorylated; Fig. 4b), but not in +N conditions (that is, when EIIA^Ntr^ is less phosphorylated; Fig. 4b). Furthermore, (p)ppGpp accumulated in *spoT*_*D81G Y323A*_ P*xylX::relA-FLAG* cells even in +N conditions (Fig. 7d), supporting again that the D81G mutation abolishes the hydrolase activity of SpoT. Finally, (p)ppGpp became undetectable in *spoT*_*Y323A*_ P*xylX::relA-FLAG* strains harbouring *ptsN*_*H66A*_ or Δ*ptsP* allele (Fig. 7d), indicating that SpoT hydrolase activity is completely unlocked when EIIA^Ntr^ is unphosphorylated. Altogether, these findings demonstrate that EIIA^Ntr^∼P inhibits hydrolase activity of SpoT to modulate (p)ppGpp accumulation upon nitrogen availability.

## Discussion

**Figure 8:**
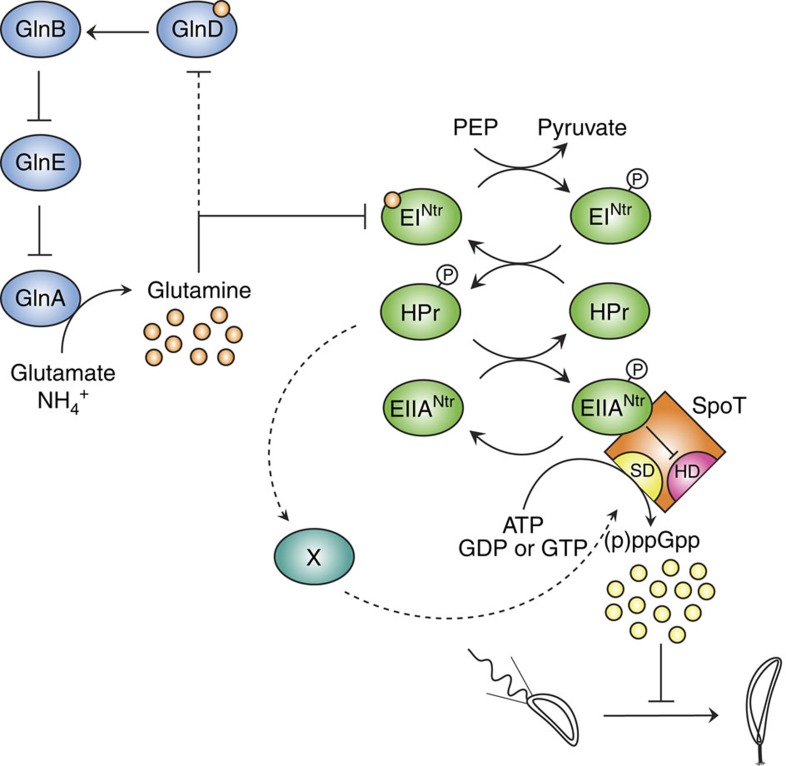
PTS^Ntr^-dependent accumulation of (p)ppGpp upon glutamine deprivation. The GlnD/GlnB/GlnE pathway (in blue) regulates glutamine homoeostasis by modulating GlnA (GS) activity. Intracellular glutamine inhibits EI^Ntr^ autophosphorylation, limiting the (p)ppGpp production in +N conditions. Note that the GlnD activity is very likely also inhibited by intracellular glutamine. On nitrogen starvation, intracellular pool of glutamine drops, relieving inhibition of EI^Ntr^ autophosphorylation and thereby increasing HPr and EIIA^Ntr^ phosphorylation levels (in green). Once phosphorylated, EIIA^Ntr^∼P interacts with SpoT to inhibit its hydrolase activity (HD), whereas HPr∼P regulates indirectly SpoT synthetase activity (SD). This dual control of SpoT by HPr∼P and EIIA^Ntr^∼P leads to (p)ppGpp accumulation, which in turn delays the G1-to-S and swarmer-to-stalked cell transition.

Adaptation to starvation conditions requires sophisticated regulatory mechanisms that sense an external stimulus and translate it into an internal molecular response. In this report, we uncovered how *Caulobacter* copes with nitrogen starvation by triggering (p)ppGpp accumulation (Fig. 8), which in turn will control the cell cycle and development by extending the G1/swarmer phase^14^,^16^. Increasing the time spent in the non-replicative (G1), motile phase reflects the adaptation of *Caulobacter* cells to their natural environment, that is, freshwater in which nutrients can rapidly be limiting^3^,^4^. Interestingly, G1 arrest also occurs during the intracellular trafficking of *Brucella abortus*, and on nitrogen and carbon starvation in *Sinorhizobium meliloti*^25^,^26^. In addition, the G1 block encountered by *S. meliloti* cells starved for nitrogen and carbon is also dependent on (p)ppGpp^26^,^27^. Therefore, (p)ppGpp-dependent mechanisms delaying DNA replication initiation could be a common feature used by α-proteobacteria in response to harsh conditions such as infection or starvation.

As previously suggested in the literature^5^, our data indicate that stalked cells are able to complete replication upon nitrogen starvation, supporting that only swarmer cells are responsive to nitrogen depletion. Indeed, even if the speed of chromosome duplication is slowed down in nitrogen-starved conditions, the stalked cell seems to be unable to stop ongoing DNA replication (Supplementary Fig. 1f). In contrast, the swarmer cell can avoid DNA replication initiation in the same conditions (Supplementary Fig. 1a–e). One reason for this difference could be that initiating DNA replication without enough nitrogen supplies would ultimately be detrimental to the cells. In support of this, we showed that deletion of *spoT* is deleterious in a Δ*glnD* background, since Δ*glnD* Δ*spoT* strain displays a strong growth defect (Fig. 3b). This result highlights the importance for *Caulobacter* swarmer cells to delay DNA replication until reaching a critical intracellular nitrogen pool.

Our data established that glutamine deprivation constitutes the intracellular signal perceived by the cell in response to nitrogen starvation and is sufficient to mediate (p)ppGpp accumulation (Fig. 8). Intracellular glutamine concentration is known to vary in bacteria, up to 10-fold depending on nitrogen availability^28^. As a consequence, monitoring intracellular glutamine concentration is an efficient strategy to evaluate nitrogen availability, and subsequently adjust nitrogen assimilation. In *E. coli*, the uridylyltransferase GlnD is known to directly sense the intracellular glutamine pool, and according to it, to modify uridylylation level of regulatory PII proteins (GlnB and GlnK), which in turn will adapt nitrogen metabolism. For instance, in the absence of glutamine, GlnD will increase ammonium transport, as well as the expression and activity of the glutamine synthetase. Intriguingly, three GlnA paralogs are encoded into the genome of *C. crescentus*, suggesting a functional redundancy, and the presence of multiple glutamine synthetase is conserved in several α-Proteobacteria^29^. Even though we showed that only the glutamine synthetase encoded by *glnA* is necessary for assimilating ammonium in complex and minimal media, we do not exclude that the two other paralogs (GlnA_2_ and GlnA_3_) display a glutamine synthetase activity under specific growth conditions that remain to be determined. Glutamine synthetase activity has been shown to promote growth of the obligatory intracellular α-proteobacterium *Ehrlichia chaffeensis* inside human cells^30^. Moreover, this successful intracellular growth of *E. chaffeensis* promoted by the glutamine pool was accompanied by a rapid degradation of CtrA, a cell cycle regulator known to inhibit DNA replication initiation in several α-proteobacteria^7^,^31^. Furthermore, the CtrA level in *Caulobacter* cells was shown to be maintained upon nitrogen starvation^5^, and even increased upon (p)ppGpp accumulation^16^. These observations suggest that the asymmetrically dividing α-proteobacteria might use glutamine as a metabolic cue for nitrogen availability that controls the cell cycle thanks to (p)ppGpp alarmone. It would be interesting to check if the PTS^Ntr^ system is used by other α-proteobacteria to relay nitrogen starvation (glutamine deprivation) to (p)ppGpp production and subsequent G1 arrest.

Only a few mechanisms triggering (p)ppGpp accumulation in nutrient-limiting conditions have so far been deciphered at the molecular level^8^. When *E. coli* cells are starved for amino acids, the (p)ppGpp synthetase RelA is directly activated by ribosomes whose A site is occupied by an uncharged tRNA^32^, whereas the bifunctional (p)ppGpp synthetase/hydrolase SpoT is regulated by an acyl carrier protein in response to fatty acid starvation^33^,^34^. In this report, we discovered a new molecular mechanism stimulating (p)ppGpp accumulation in response to nutrient starvation. This mechanism involves the PTS^Ntr^ system as an important metabolic sensor that translates a glutamine deprivation signal into a (p)ppGpp accumulation signal. Our data suggest that EIIA^Ntr^∼P directly reduces the hydrolase activity of SpoT, while HPr∼P indirectly activates (p)ppGpp production upon nitrogen starvation (Fig. 8). Historically, the PTS system was discovered as a phosphorylation cascade involved in the regulation of sugar uptake and carbon catabolite repression^21^,^35^. Afterwards, a second phosphotransferase system (PTS^Ntr^) was proposed to be connected to nitrogen metabolism but this connection remained poorly described^21^. The direct inhibition of EI^Ntr^ autophosphorylation by glutamine observed in *E. coli* and *S. meliloti*^22^,^36^, as well as now in *C. crescentus* (Fig. 4), reinforces the idea that nitrogen constitutes a signal for PTS^Ntr^ systems. The fact that the GAF domain, highly conserved in all EI^Ntr^ proteins (Supplementary Fig. 4b), is required for binding glutamine suggests that the glutamine-dependent control of EI^Ntr^ phosphorylation might be a common feature in PTS^Ntr^ system.

In contrast to its EIIA paralog, the EIIA^Ntr^ component is not associated with permeases, but rather carries out regulatory functions, by interacting with its target(s). For example, the unphosphorylated form of EIIA^Ntr^ inhibits pyruvate dehydrogenase activity in *Pseudomonas putida* by interacting with the E1 subunit^37^. Our work constitutes so far the first example of regulatory functions mediated by the phosphorylated form of EIIA^Ntr^ (EIIA^Ntr^∼P). Indeed, our results support the conclusion that only the phosphorylated form of EIIA^Ntr^ interacts with SpoT to inhibit its hydrolase activity. This is further supported by the fact that EI^Ntr^ and SpoT are found in the same protein complex during stationary phase^38^. Interestingly, a direct interaction between the non-phosphorylated form of EIIA^Ntr^ and SpoT has been recently found in the β-proteobacterium *Ralstonia eutropha* by BTH but no function was assigned for this connection^39^. This differential interaction between phosphorylated or non-phosphorylated form EIIA^Ntr^ and SpoT illustrates the evolutionary plasticity of PTS^Ntr^ components with their targets.

Besides EIIA^Ntr^∼P, we know that phosphorylated HPr also controls (p)ppGpp accumulation on nitrogen starvation, but how this regulation works at the molecular level remains an open question. HPr∼P could interact with an unknown factor (X in Fig. 8), which in turn could modulate the abundance of SpoT or activate its synthetase activity, to subsequently increase the global (p)ppGpp pool. Although we have now uncovered the pathway that stimulates (p)ppGpp accumulation in response to nitrogen starvation, understanding how (p)ppGpp affects the G1-to-S transition at the molecular level will be a challenge for future work.

## Methods

### Bacterial strains and growth conditions

Oligonucleotides, strains and plasmids used in this study are listed in Supplementary Tables 1, 2 and 3, altogether with construction details provided in the Supplementary Methods. *E. coli* Top10 was used for cloning purpose, and grown aerobically in Luria–Bertani (LB) broth (Sigma)^40^. Electrocompetent cells were used for transformation of *E. coli.* All *Caulobacter crescentus* strains used in this study are derived from the synchronizable wild-type strain NA1000, and were grown in PYE or synthetic M2 (20 mM PO_4_^3−^, 9.3 mM NH_4_^+^; +N) or P2 (20 mM PO_4_^3−^; −N) supplemented with 0.5 mM MgSO_4_, 0.5 mM CaCl_2_, 0.01 mM FeSO_4_ and 0.2% glucose (M2G or P2G, respectively) media at 28–30 °C. Glutamine (Q) was used at a final concentration of 9.3 mM. Growth was monitored by following the OD (600 nm) during 24 h, in an automated plate reader (Bioscreen C, Lab Systems) with continuous shaking at 30 °C. Genes expressed from the inducible *vanA* promoter (P_*vanA*_) was induced with 0.5 mM vanillate. Generalized transduction was performed with phage ФCr30 according to the procedure described in ref. 41. Motility was monitored on PYE swarm (0.3% agar) plates. Area of the swarm colonies were quantified with ImageJ software as described previously in ref. 42. Motility screen was performed on PYE swarm (0.3% agar) plates supplemented with glutamine (9.3 mM) during 3–4 days at 30 °C. Genomic DNA of the candidates was then sequenced by the Illumina sequencing method (Beckman Coulter Genomics). Transpositional screen was performed with *himar1* transposons on PYE plates as previously described in ref. 43. The exact positions of three *himar1* insertion sites into the *ptsP* locus (Fig. 3a) have been determined by semi-arbitrary PCR. The presence of *himar1* transposons into the *ptsP* locus was checked by PCR with primers (926 and 927) hybridizing upstream and downstream of *ptsP*. For *E. coli*, antibiotics were used at the following concentrations (μg ml^−1^; in liquid/solid medium): ampicillin (50/100), kanamycin (30/50), oxytetracycline (12.5/12.5). For *C. crescentus*, media were supplemented with kanamycin (5/20), oxytetracycline (1/2.5) where appropriate. The doubling time of *Caulobacter* strains was calculated in exponential phase (OD_660_: 0.2–0.5) using D=(ln(2)·(T_(B)_−T_(A)_))/(ln(OD_660(B)_)−ln(OD_660(A)_)) and normalized according to the wild-type strain. *E. coli* S17–1 and *E. coli* MT607 helper strains were used for transferring plasmids to *C. crescentus* by bi- and tri-parental mating, respectively. In-frame deletions were created by using pNPTS138-derivative plasmids and by following the procedure described previously in ref. 44.

### Flow cytometry analysis

DNA content was measured using fluorescence-activated cell sorting (FACS). Cells were fixed in ice-cold 70% ethanol. Fixed samples were then washed twice in FACS staining buffer (10 mM Tris pH 7.2, 1 mM EDTA, 50 mM NaCitrate, 0.01% Triton X-100) containing 0.1 mg ml^−1^ RNaseA and incubated at room temperature (RT) for 30 min. Cells were then collected by centrifugation for 2 min at 8,000*g*, resuspended in 1 ml FACS staining buffer containing 0.5 μM Sytox Green Nucleic acid stain (Life Technologies), and incubated at RT in the dark for 5 min. Samples were analysed in flow cytometer (FACS Calibur, BD Biosciences) at laser excitation of 488 nm. At least 1 × 10^4^ cells were recorded in triplicate for each experiment. Gate for cells in G1 phase was determined with a sample of wild-type cells treated with Rifampicin (2.5 μg ml^−1^) for 3 h. Percentage of gated G1 cells of each strain was then normalized using gated G1 cells of the wild-type strain as reference.

### Synchronization of cells

For synchrony, cells were grown in 200 ml of PYE (OD_660_ ∼0.8), collected by centrifugation for 15 min at 6,000*g*, 4 °C; resuspended in 60 ml of ice-cold 20 mM phosphate (PO_4_^3−^) buffer and combined with 30 ml of Ludox LS Colloidal Silica (30%; Sigma-Aldrich)^45^. Cells resuspended in Ludox was centrifuged for 40 min at 9,000*g*, 4 °C. Swarmer cells, corresponding to the bottom band, were isolated, washed twice in ice-cold PO_4_^3−^ buffer and finally resuspended in prewarmed PYE media for growth at 30 °C. Samples were collected every 15 min for western blot, microscopy and FACS analyses.

### Light and fluorescent microscopy

All strains were imaged during exponential growth phase after immobilization on 1% agarose pads^41^. Microscopy was performed using Axioskop microscope (Zeiss), Orca-Flash 4.0 camera (Hamamatsu) and Zen 2012 software (Zeiss). Images were processed with ImageJ. Supplementary Movie 1 was done with Debut Video Capture Software.

### Detection of intracellular (p)ppGpp levels

(p)ppGpp levels were visualized as described previously in ref. 13. Briefly, strains were grown overnight in PYE and then diluted for a second overnight culture in M5GG (low-phosphate medium M5G supplemented with 1 mM glutamate). Then, cells were diluted a second time in M5GG and grown for 3 h to reach an OD_660_ of 0.4. Cells were split into two parts and washed twice with P5G-labelling buffer (M5G without NH_4_^+^ and with 12.2 mM NaCl and 3.9 mM KCl instead of Na_2_HPO_4_ and KH_2_PO_4_). In all, 1 ml of cells were then resuspended in 225 μl of P5G-labelling (−N) or M5G-labelling (+N) supplemented with 25 μl of KH_2_^32^PO_4_ at 100 μCi ml^−1^ and incubated for 2 h with shaking (450 r.p.m.) at 30 °C. Then, samples were extracted with an equal volume of 2 M formic acid, placed on ice for 30 min and then stored overnight at −20 °C. All cell extracts were pelleted at 18,000*g* for 3 min and 6 × 2 μl of supernatant was spotted onto a polyethyleneimine plate (Macherey-Nagel). Polyethyleneimine plates were then developed in 1.5 M KH_2_PO_4_ (pH 3.4) at RT. Finally, TLC plates were imaged on a MS Storage Phosphor Screen (GE Healthcare) and analysed with Cyclone Phosphor Imager (PerkinElmer). For hydrolase experiments (Fig. 7d), cells were incubated 1 h in P5G supplemented with xylose (0.1%). Then, cells were washed twice with P5G-labelling and resuspended in P5G-labelling (−N) supplemented with KH_2_^32^PO_4_, xylose (0.1%) and with (+N) or without (−N) glutamine (9.3 mM).

### BTH assays

BTH assays were performed as described previously in refs 23 and 42. Briefly, 2 μl of MG1655 *cyaA::frt* (RH785), MG1655 *cyaA::frt* Δ*npr* (RH2122), MG1655 *cyaA::frt* Δ*ptsP* Δ*ptsI* (RH2124) strains expressing T18 and T25 fusions were spotted on MacConkey Agar Base plates supplemented with ampicillin, kanamycin, maltose (1%) and IPTG (1 mM) and incubed for 3–4 days at 30 °C. All proteins were fused to T25 at their N-terminal extremity (pKT25) or to the T18 at their N- (pUT18C) or C-terminal (pUT18) extremity. BTH assays in both directions (T25-X with T18-Y or T25-Y with T18-X) gave similar results.

The β-galactosidase assays were performed as described in ref. 46. Briefly, 50 μl *E. coli* BTH strains cultivated overnight at 30 °C in LB medium supplemented with kanamycin, ampicillin and IPTG (1 mM) were resuspended in 800 μl of Z buffer (60 mM Na_2_HPO_4_, 40 mM NaH_2_PO_4_, 10 mM KCl, 1 mM MgSO_4_) and lysed with chloroform. After the addition of 200 μl ONPG (4 mg ml^−1^), reactions were incubated at 30 °C until colour turned yellowish. Reactions were then stopped by the addition of 500 μl of 1 M Na_2_CO_3_, and absorbance at 420 nm was measured. Miller units are defined as (OD_420_ × 1,000)/(OD_590_ × t × v), where, ‘OD_590_' is the absorbance at 590 nm of the cultures before the β-galactosidase assays, ‘t' is the time of the reaction (min) and ‘v' is the volume of cultures used in the assays (ml). All the experiments were performed with three biological replicates and Miller units of the T25-X T18-ZIP combination were used as a blank and substracted.

### Immunoblot analysis

Immunoblot analyses were performed as described in ref. 46, with the following primary antibodies: α-Flagellin (1:5,000; ref. 47), α-FLAG (1:5,000; Stratagene), α-MreB (1:5,000; ref. 46) and secondary antibodies: 1:10,000 anti-mouse (for α-FLAG) or 1:7,500 anti-rabbit (for all the others) linked to peroxidase (GE Healthcare), and visualized thanks to Western Lightning Plus-ECL chemiluminescence reagent (Biorad) and ImageQuant LAS400 (GE Healthcare).

### Preparation of fractions containing EI^Ntr^ or EI^Ntr^_L83Q_

Fractions containing EI^Ntr^ or EI^Ntr^_L83Q_ proteins were purified from NA1000 and NA1000 *ptsP*_*L83Q*_ (RH1748) strains, respectively. *C. crescentus* strains were grown in 150 ml PYE liquid media (OD_660_ ∼0.7), collected by centrifugation for 15 min at 6,000*g*, 4 °C, washed by ice-cold 20 mM phosphate buffer and then resuspended in 5 ml ice-cold phosphate buffered-saline containing 0.05% Triton X-100, complete EDTA-free anti-proteases, 20 mg ml^−1^ lysozyme, 10 U ml^−1^ DNase I. Cells were first lysed by sonication, then zirconium beads were added and cells were disrupted by Fastprep cycles (5 × 20 s). Lysates were pelleted at 15 000g at 4 °C and then resuspended in 500 μl of buffer containing 25 mM Tris/HCl pH 7.5, 10 mM MgCl_2_, 1 mM DTT.

### Autophosphorylation levels of EI^Ntr^ and EI^Ntr^_L83Q_

[^32^P]PEP was prepared enzymatically as described previously in ref. 48. Briefly, 50 μl reaction solution containing 100 mM triethylamine/HCl pH 7.6, 15 mM KCl, 3 mM MgCl_2_, 165 μM PEP, 1 mM pyruvate, 5 μM ATP, 60 μCi [γ-^32^P]-ATP (5,000 Ci mmol^−1^) and 40 units of pyruvate kinase (Sigma) were incubated at 30 °C for 2 h. Phosphorylation assays were performed in 20 μl of solution containing 10 μl of proteins extract (containing EI^Ntr^ or EI^Ntr^_L83Q_), 25 mM Tris/HCl pH 7.5, 10 mM MgCl_2_, 1 mM DTT, glutamine (0, 2, 5 or 10 mM) and 0.5 μl of [^32^P]PEP solution at 37 °C for 30 min. Then, 5 μl of 5 × SDS-PAGE loading buffer were added to the samples. Proteins were subjected to electrophoresis in a 10% SDS-polyacrylamide gel. SDS-polyacrylamide gels were then dried and imaged on a MP Phosphor system (Packard) and analysed with Cyclone Phosphor Imager (PerkinElmer). Analysis of radioactive spots reveals three bands at different size (∼50, 80 and 90 kDa). The band corresponding to ∼80 kDa, absent in protein extracts from the Δ*ptsP* strain, was determined as EI^Ntr^ or EI^Ntr^_L83Q_.

### *In vivo* phosphorylation of EIIA^Ntr^

Strains containing pXMCS2*-3FLAG-ptsN* were grown overnight in PYE supplemented with kanamycin and then diluted for a second overnight culture in M5GG (low-phosphate medium M5G supplemented with 1 mM glutamate) supplemented with kanamycin. Then, cells were diluted in M5GG with or without xylose (0.1%), and grown for 3 h to reach an OD_660_ of 0.5. In all, 1 ml of cells were washed twice with P5G-labelling buffer (M5G without NH_4_^+^ and with 12.2 mM NaCl and 3.9 mM KCl instead of Na_2_HPO_4_ and KH_2_PO_4_). Cells were then resuspended in 225 μl of P5G-labelling, P5X-labelling (xylose 0.1%) or P5XQ-labelling (xylose 0.1%, glutamine 9.3 mM) supplemented with 25 μl of KH_2_^32^PO_4_ at 100 μCi ml^−1^ and incubated for 2 h with shaking (450 r.p.m.) at 30 °C. Samples were collected for 2 min at 12,000 r.p.m., resuspended in 50 μl of lysis buffer (50 mM Tris pH 7.0, 80 mM EDTA, 150 mM NaCl, 4% Triton X-100) and incubated for 2 min at 4 °C. Then, 900 μl of low-salt buffer (50 mM Tris pH 7.0, 100 mM NaCl, 50 mM EDTA, 2% Triton X-100) were added and samples were collected for 15 min at 4 °C. Supernatants were mixed with anti-FLAG M2 magnetic beads (Sigma) previously washed three times with TBS buffer and twice with low-salt buffer. Samples were incubated on a rotating shaker for 90 min at 4 °C, and beads were washed once with cold low-salt buffer and twice with cold high-salt buffer (50 mM Tris pH 7.0, 500 mM NaCl, 50 mM EDTA, 0.1% Triton X-100). Magnetic beads were then resuspended in 20 μl of 3 × SDS loading buffer and 5 μl of 0.5 mg ml^−1^ of 3FLAG peptide (Sigma) were added to each sample. After 10 min incubation with shaking (1,300 r.p.m.), proteins were subjected to electrophoresis in a 12% SDS-polyacrylamide gel. SDS-polyacrylamide gels were then dried and imaged on a MP Phosphor system (Packard) and analysed with Cyclone Phosphor Imager (PerkinElmer). Band intensities were quantified with ImageJ software by using the WT (−Xyl, −N) as the background.

## Additional information

**How to cite this article:** Ronneau, S. *et al*. Phosphotransferase-dependent accumulation of (p)ppGpp in response to glutamine deprivation in *Caulobacter crescentus*. *Nat. Commun.* 7:11423 doi: 10.1038/ncomms11423 (2016).

## Supplementary Material

Supplementary Information Supplementary Figures 1-10, Supplementary Tables 1-3, Supplementary Methods and Supplementary References.

Supplementary Movie 1 Motility of *Caulobacter crescentus* wild-type (left), Δ*spoT* (middle) and *spoT_D81G_* (right) cells in complex PYE medium.
