# Supplementary material for: Phosphotransferase-dependent accumulation of (p)ppGpp in response to glutamine deprivation in Caulobacter crescentus
Source: Nat Commun. 2016 Apr 25;7:11423. doi: 10.1038/ncomms11423 (PMC4848567; doi:10.1038/ncomms11423)
Supplement: Supplementary Information — Supplementary Figures 1-10, Supplementary Tables 1-3, Supplementary Methods and Supplementary References. [file ncomms11423-s1.pdf]

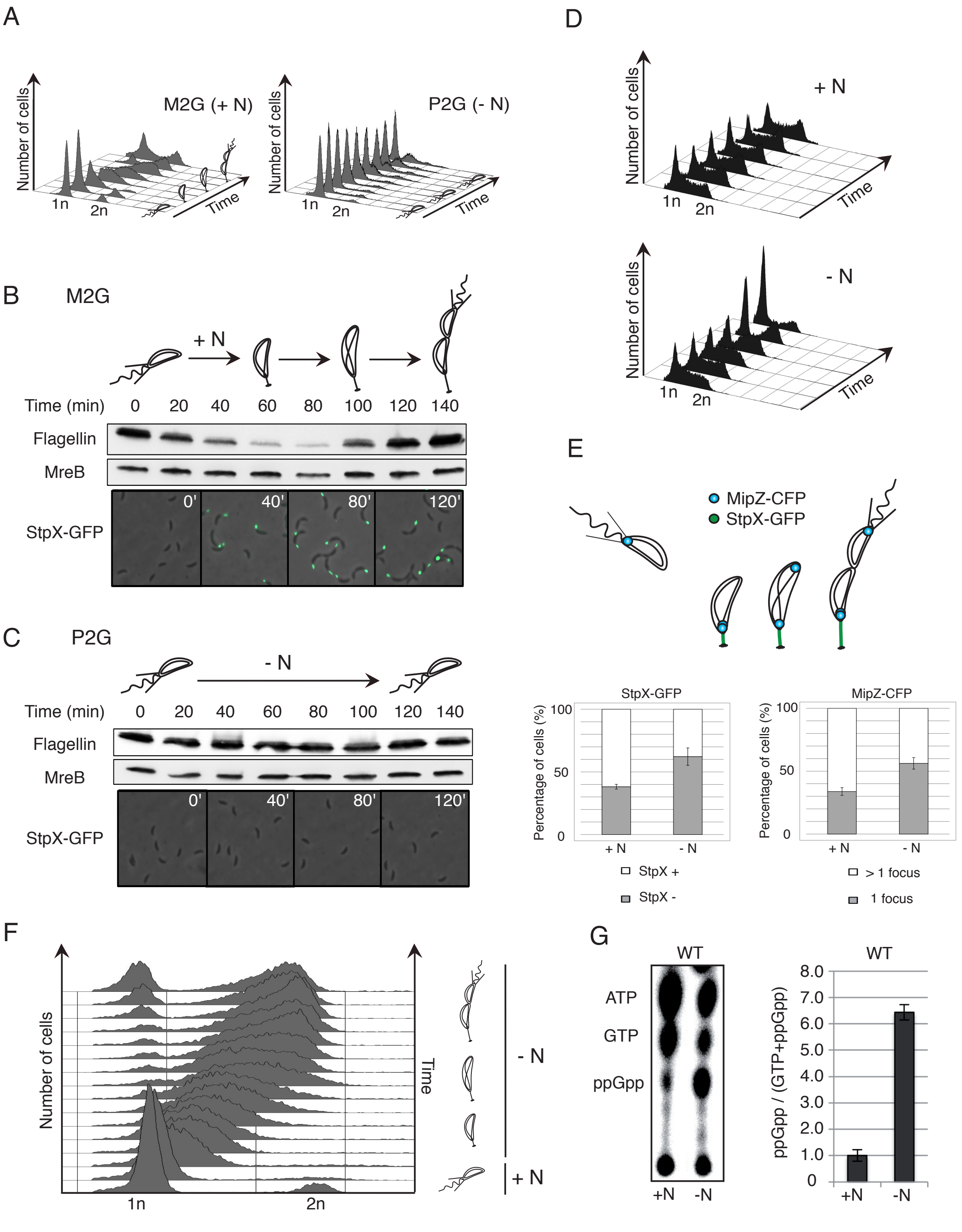
A
M2G (+ N)
P2G (- N)
Number of cells
1n
2n
Time
B
M2G
+ N
Time (min) 0 20 40 60 80 100 120 140
Flagellin
MreB
StpX-GFP
0' 40' 80' 120'
C
P2G
- N
Time (min) 0 20 40 60 80 100 120 140
Flagellin
MreB
StpX-GFP
0' 40' 80' 120'
D
+ N
- N
Number of cells
1n
2n
Time
E
MipZ-CFP
StpX-GFP
StpX-GFP
MipZ-CFP
Percentage of cells (%)
100
50
0
+ N
- N
StpX +
StpX -
> 1 focus
1 focus
F
Number of cells
Time
- N
+ N
1n
2n
G
WT
ATP
GTP
ppGpp
+N
-N
ppGpp / (GTP+ppGpp)
8.0
7.0
6.0
5.0
4.0
3.0
2.0
1.0
0

Supplementary Figure 1. *Caulobacter crescentus* accumulates G1/swarmer cells and (p)ppGpp upon nitrogen starvation.

(**A**) Swarmer cells cannot replicate DNA upon nitrogen starvation. Flow cytometry analysis was used to determine DNA content throughout the cell cycle of WT (RH50) grown in M2G (+N) or P2G (-N) media. Samples were withdrawn every 20 min from a synchronized population of WT (RH50) grown in M2G or P2G.

(**B-C**) Swarmer cells cannot differentiate into stalked cells upon nitrogen starvation. Immunoblot and fluorescence microscopy analyses were used to respectively determine the relative abundance of proteins (Flagellin and MreB), and localization of StpX-GFP throughout the cell cycle of *C. crescentus* strains grown in M2G (+N) or P2G (-N) media. Proteins were extracted every 20 min from a synchronized population of WT (RH50) grown in M2G (**B**) or P2G (**C**). Phase contrast and fluorescence micrographs were taken every 40 min from a synchronized population of NA1000 *stpX::stpX-gfp* (RH507) grown in M2G (B) or P2G (C).

(**D-E**) Swarmer cells can neither initiate DNA replication nor differentiate into stalked cell upon nitrogen starvation. (**D**) Flow cytometry analysis was used to determine DNA content in asynchronous population of WT (RH50) grown for 6 hours in M2G (+N) or P2G (-N) media. Samples were withdrawn every hour from a synchronized population of WT (RH50) grown in M2G or P2G. (**E**) Fluorescence microscopy to determine localization of StpX-GFP and MipZ-CFP in asynchronous population of WT (RH50) grown for 6 hours in M2G (+N) or P2G (-N) media. G1/swarmer cells (without StpX-GFP signal or with only 1 focus of MipZ-CFP) were counted and normalized (grey columns) to the total number of cells (100%).

(**F**) Synchronized stalked cells are able to complete DNA replication despite the absence of nitrogen source. Samples were withdrawn every 20 min from a synchronized population of WT (RH50) first grown in M2G (+N) for the first 40 min (to allow the differentiation of swarmer cells into stalked cells) and then in P2G (-N).

(**G**) *C. crescentus* accumulates (p)ppGpp upon nitrogen starvation. Intracellular levels of (p)ppGpp detected by TLC after nucleotides extraction of WT (RH50) grown in nitrogen-replete (+N) or -deplete (-N) conditions. Error bars = SD, n = 3.

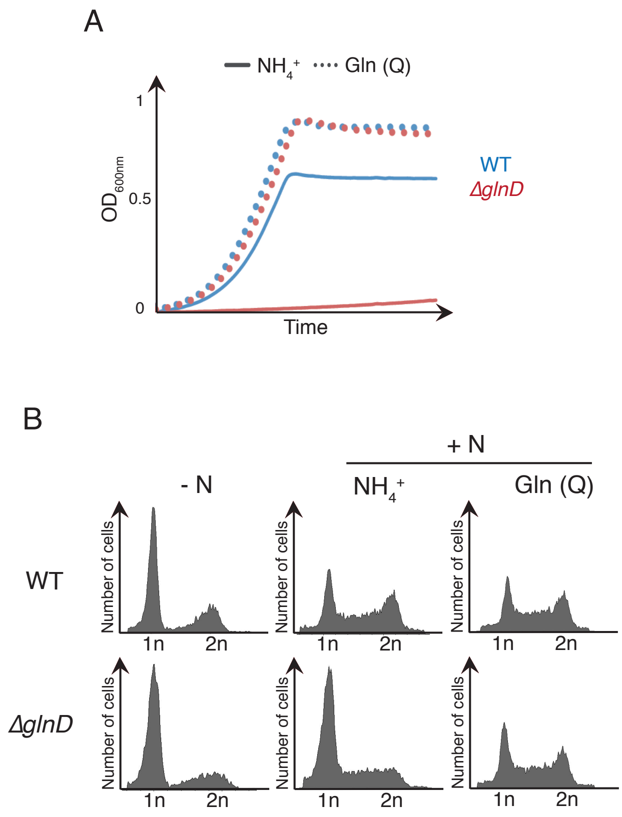

Supplementary Figure 2. Glutamine auxotrophy displayed by Δ*glnD* responsible for G1/swarmer cells accumulation displayed by *ΔglnD*.

(**A**) Growth of WT (RH50) and Δ*glnD* (RH577) in minimal media containing NH_4^+ (M2G) or glutamine (P2GQ) as the only nitrogen source. (**B**) Flow cytometry analysis to determine DNA content in asynchronous population of WT (RH50) and Δ*glnD* (RH577) in minimal media without any nitrogen source (P2G) or with NH_4^+ (M2G) or glutamine (P2GQ) as the only nitrogen source.

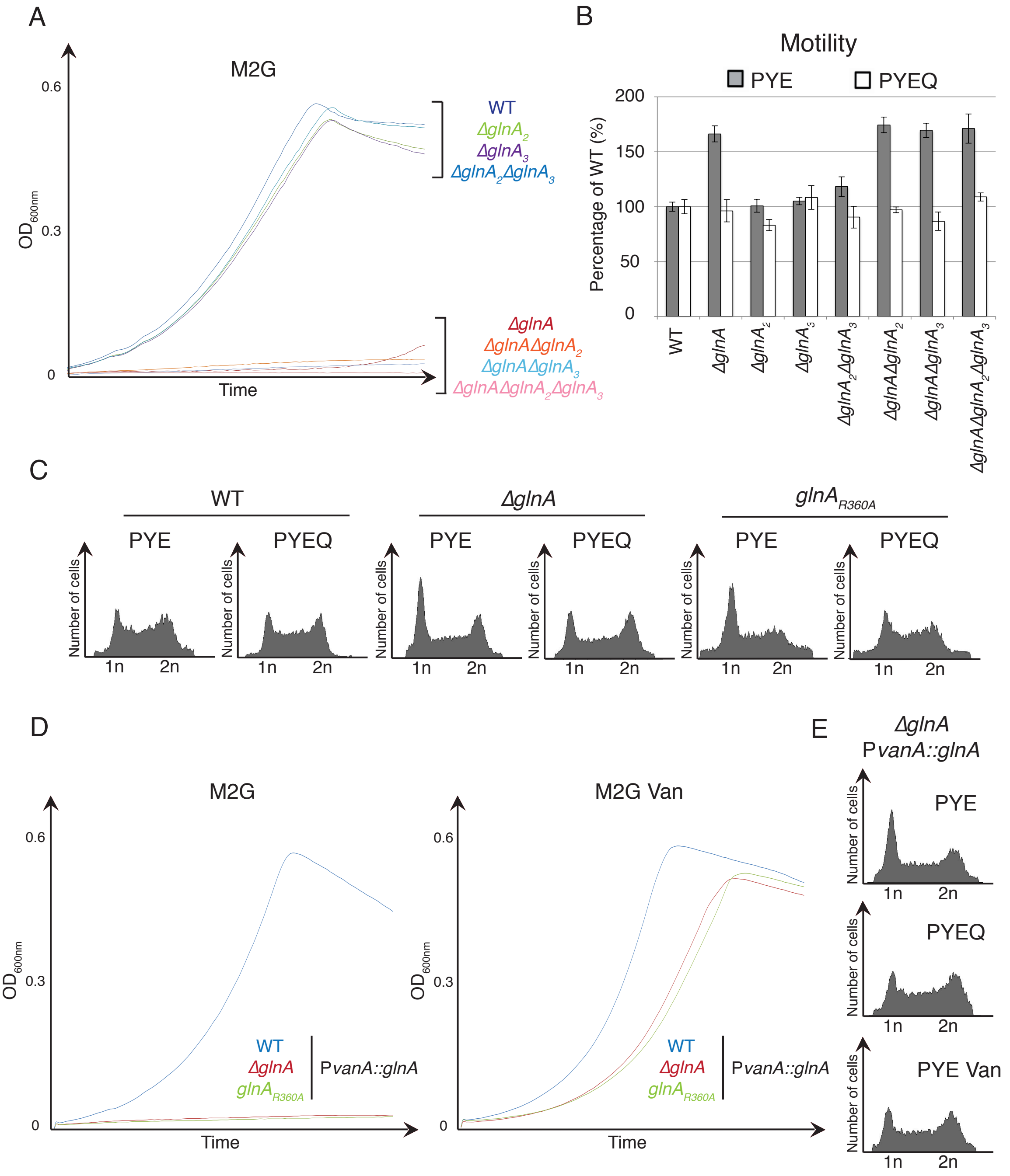
A
M2G
0.6
0.3
0
OD_{600nm}
Time
WT
ΔglnA2
ΔglnA3
ΔglnA2ΔglnA3
ΔglnA
ΔglnAΔglnA2
ΔglnAΔglnA3
ΔglnAΔglnA2ΔglnA3
B
Motility
PYE
PYEQ
Percentage of WT (%)
200
150
100
50
0
WT
ΔglnA
ΔglnA2
ΔglnA3
ΔglnA2ΔglnA3
ΔglnAΔglnA2
ΔglnAΔglnA3
ΔglnAΔglnA2ΔglnA3
C
WT
ΔglnA
glnAR360A
PYE
PYEQ
Number of cells
1n
2n
D
M2G
M2G Van
OD_{600nm}
0.6
0.3
0
Time
WT
ΔglnA
glnAR360A
PvanA::glnA
E
ΔglnA
PvanA::glnA
PYE
PYEQ
PYE Van
Number of cells
1n
2n

Supplementary Figure 3. GlnA is the only glutamine synthetase required to assimilate ammonium in *Caulobacter crescentus* grown in minimal and complex media.

(**A-C**) Loss-of-function *glnA* mutants are auxotrophic for glutamine, cannot grow in minimal medium (M2G) and increase G1/swarmer cells proportion in complex medium (PYE). (**A**) Growth in minimal medium and (**B**) motility on PYE swarm agar plates without (PYE) or with glutamine (PYEQ) of WT (RH50), Δ*glnA* (RH772) Δ$glnA_2$ (RH876), Δ$glnA_3$ (RH877), Δ$glnA_2$ $glnA_3$ (RH878), Δ$glnA_2$ *glnA* (RH970), Δ$glnA_3$*glnA* (RH971) and Δ$glnA_2$ $glnA_3$ *glnA* (RH879). (**C**) Flow cytometry analysis to determine DNA content in asynchronous population of WT (RH50) and Δ*glnA* (RH772) and $glnA_{R360A}$ (RH1632) grown in complex medium (PYE) supplemented with glutamine (PYEQ).

(**D-E**) Loss-of-function *glnA* mutants can be complemented by expressing *glnA in trans*. (**D**) Growth of WT (RH50) and Δ*glnA* (RH772) and $glnA_{R360A}$ (RH1632) harbouring P_{vanA}::*glnA* plasmid (pHR533) in minimal medium (M2G) supplemented with 0.5 mM vanillate (M2G Van). (**E**) Flow cytometry analysis to determine DNA content in asynchronous population of WT (RH50), Δ*glnA* (RH772) and $glnA_{R360A}$ (RH1632) harbouring P_{vanA}::*glnA* plasmid (pHR533) in complex medium (PYE) supplemented with 0.5 mM vanillate (PYE Van) or glutamine (PYEQ).

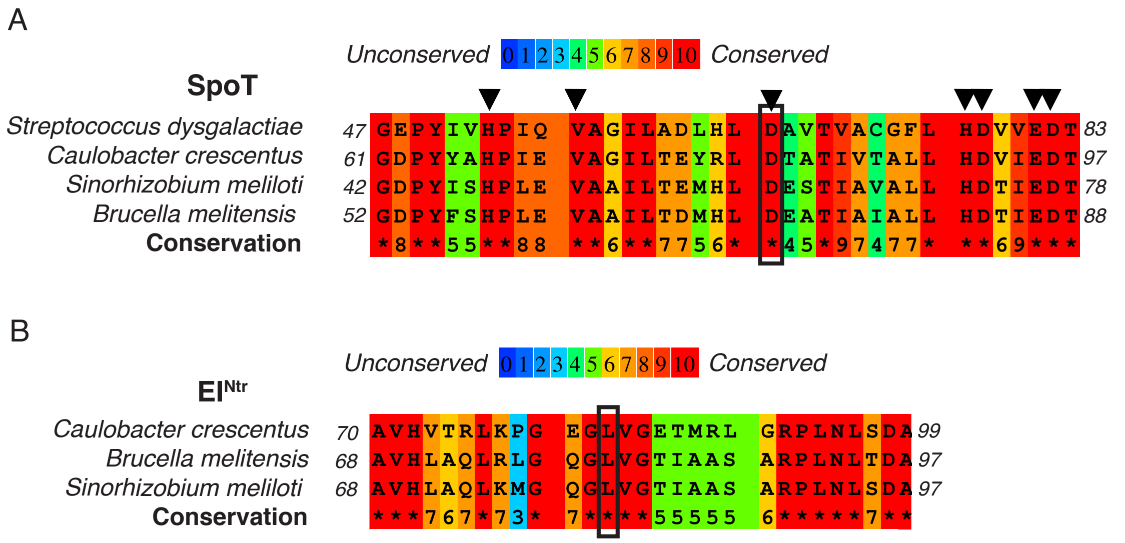

Supplementary Figure 4. The gain-of-function mutations isolated in SpoT and EI^{Ntr} reside in highly conserved regions.

(**A**) Aspartate 81 of SpoT$_{Cc}$ is highly conserved in proteobacteria. Black arrowheads indicate mutations described to abolish hydrolase activity of SpoT in *Steptococcus dysgalactiae* subsp. *equisimilis* [1].

(**B**) Leucine 83 of EI$^{Ntr}_{Cc}$ is highly conserved in α-proteobacteria.

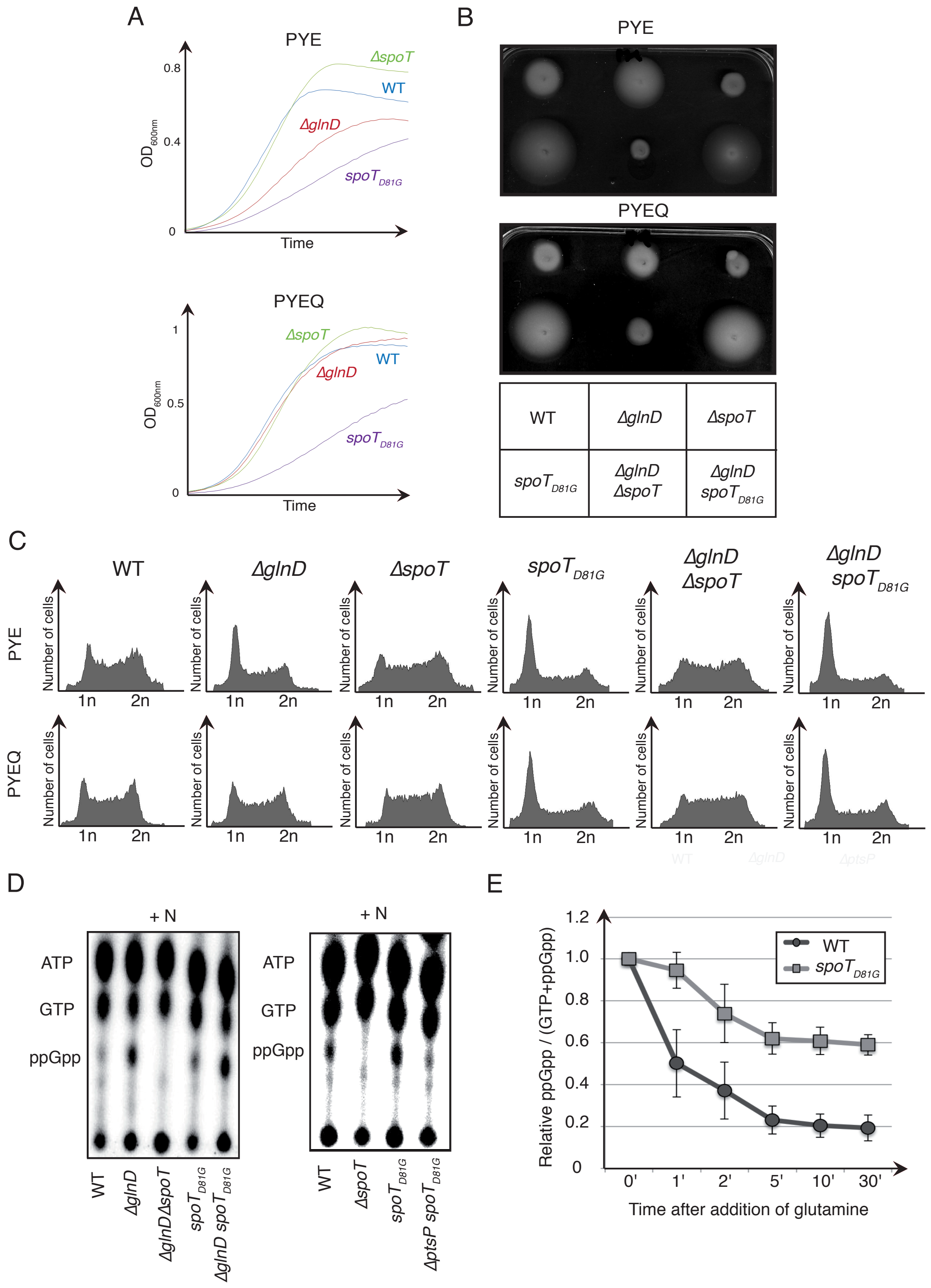
A
PYE
ΔspoT
WT
ΔglnD
spoT D81G
0.8
0.4
0
OD600nm
Time
PYEQ
ΔspoT
WT
ΔglnD
spoT D81G
1
0.5
0
OD600nm
Time
B
PYE
PYEQ
WT
ΔglnD
ΔspoT
spoT D81G
ΔglnD ΔspoT
ΔglnD spoT D81G
C
WT
ΔglnD
ΔspoT
spoT D81G
ΔglnD ΔspoT
ΔglnD spoT D81G
PYE
PYEQ
Number of cells
1n
2n
D
+ N
ATP
GTP
ppGpp
WT
ΔglnD
ΔglnDΔspoT
spoT D81G
ΔglnD spoT D81G
+ N
ATP
GTP
ppGpp
WT
ΔspoT
spoT D81G
ΔptsP spoT D81G
E
WT
spoT D81G
1.2
1.0
0.8
0.6
0.4
0.2
0
Relative ppGpp / (GTP+ppGpp)
0'
1'
2'
5'
10'
30'
Time after addition of glutamine

Supplementary Figure 5. SpoT controls the G1/swarmer lifetime upon nitrogen starvation.

(**A-C**) The growth defect and G1/swarmer accumulation of *spoT*$_{D81G}$ cannot be compensated by exogenous source of glutamine. (**A**) Growth of WT (RH50), Δ*glnD* (RH577), Δ*spoT* (RH1755) and *spoT*$_{D81G}$ (RH1752) in complex medium without (PYE) or with glutamine (PYEQ). (**B**) Motility on swarm agar plates and (**C**) DNA content determined by flow cytometry of WT (RH50), Δ*glnD* (RH577), Δ*spoT* (RH1755), *spoT*$_{D81G}$ (RH1752), Δ*glnD* Δ*spoT* (RH1756) and Δ*glnD* *spoT*$_{D81G}$ (RH1753) grown in complex medium without (PYE) or with glutamine (PYEQ).

(**D**) PtsP is required for the SpoT-dependent (p)ppGpp accumulation upon nitrogen starvation. Intracellular levels of (p)ppGpp detected by TLC after nucleotides extraction of WT (RH50), Δ*glnD* (RH577), Δ*glnD* Δ*spoT* (RH1756), *spoT*$_{D81G}$ (RH1752), Δ*glnD* *spoT*$_{D81G}$ (RH1753), Δ*spoT* (RH1755), Δ*ptsP* *spoT*$_{D81G}$ (RH1727) grown in nitrogen-replete (+N) conditions.

(**E**) The hydrolysis of (p)ppGpp is reduced in *spoT*$_{D81G}$ in comparison to the wild-type strain. The timing of (p)ppGpp removal was determined by measuring (p)ppGpp content in WT (RH50) and *spoT*$_{D81G}$ (RH1752) after 2h in nitrogen starvation followed by addition of glutamine (T = 0). Error bars = SD, n = 2.

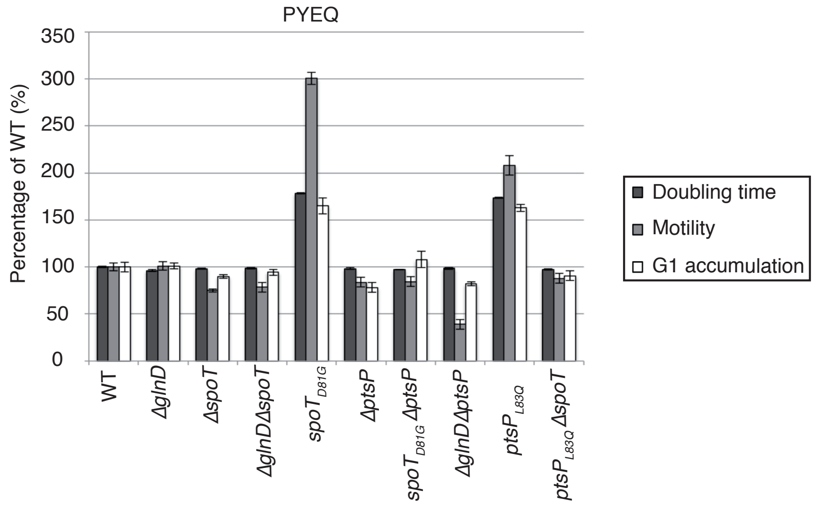

Supplementary Figure 6. The cell cycle and developmental defects of *spoT*$_{D81G}$ and *ptsP*$_{L83Q}$ cannot be compensated by addition of exogenous source of glutamine.

Growth, motility and DNA content of WT (RH50), Δ*glnD* (RH577), Δ*spoT* (RH1755), *ΔspoT ΔglnD* (RH1756), *spoT*$_{D81G}$ (RH1752), ΔptsP (RH1758), *ΔptsP spoT*$_{D81G}$ (RH1727), *ΔglnD ΔptsP* (RH1940), *ptsP*$_{L83Q}$ (RH1748) and *ptsP*$_{L83Q}$ *ΔspoT* (RH1728) were determined in complex medium supplemented with glutamine (PYEQ).

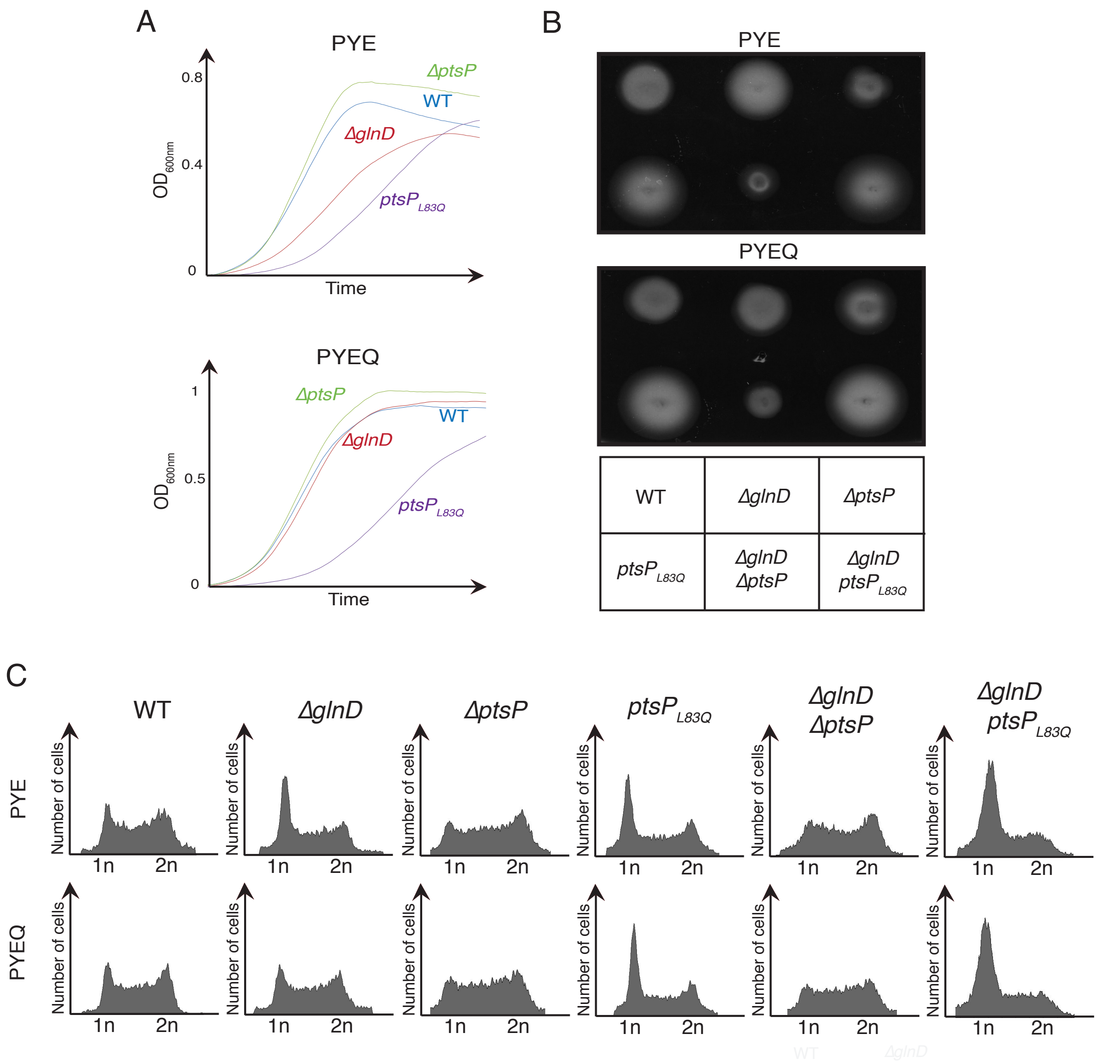

Supplementary Figure 7. EI^{Ntr} controls the G1/swarmer lifetime upon nitrogen starvation.

(**A-C**) The growth defect and G1/swarmer accumulation of *ptsP*$_{L83Q}$ cannot be compensated by exogenous source of glutamine. (**A**) Growth of WT (RH50), Δ*glnD* (RH577), Δ*ptsP* (RH1758) and *ptsP*$_{L83Q}$ (RH1748) in complex medium without (PYE) or with glutamine (PYEQ). (**B**) Motility on swarm agar plates and (**C**) DNA content determined by flow cytometry of WT (RH50), Δ*glnD* (RH577), Δ*ptsP* (RH1758), *ptsP*$_{L83Q}$ (RH1748), Δ*glnD* Δ*ptsP* (RH1940) and *ptsP*$_{L83Q}$ Δ*glnD* (RH1941) grown in complex medium without (PYE) or with glutamine (PYEQ).

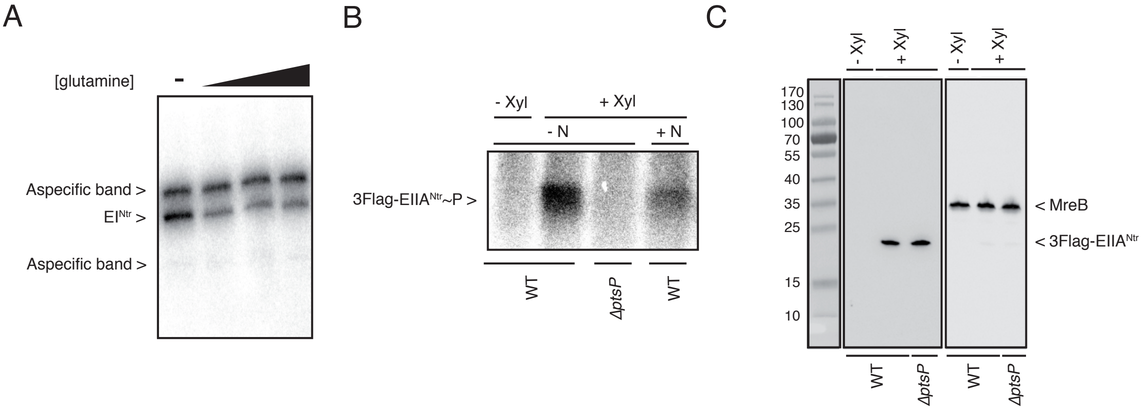

Supplementary Figure 8. Glutamine inhibits PTS^{Ntr} phosphorylation.

(**A**) Glutamine inhibits autophosphorylation of EI^{Ntr}. Autophosphorylation assays of EI^{Ntr} using [^{32}P]PEP as a phosphoryl donor in the absence or presence of increasing concentration of glutamine (0, 2, 5, 10 mM).

(**B**) The EI^{Ntr}-dependent phosphorylation of EIIA^{Ntr} is enhanced upon nitrogen starvation. *In vivo* phosphorylation assays of EIIA^{Ntr} in nitrogen-deplete (-N) or nitrogen-replete (+N) conditions supplemented with (+ Xyl) or without (- Xyl) xylose in WT or Δ*ptsP* expressing *3FLAG-ptsN* from P*xylX* promoter.

(**C**) Immunoblotting of protein samples extracted from WT and Δ*ptsP* expressing *3FLAG-ptsN* from P*xylX* promoter, incubated 3 hours in M5GG supplemented with (+ Xyl) or without (- Xyl) xylose. MreB was detected in all conditions while 3FLAG-EIIA^{Ntr} was detected only in the presence of xylose.

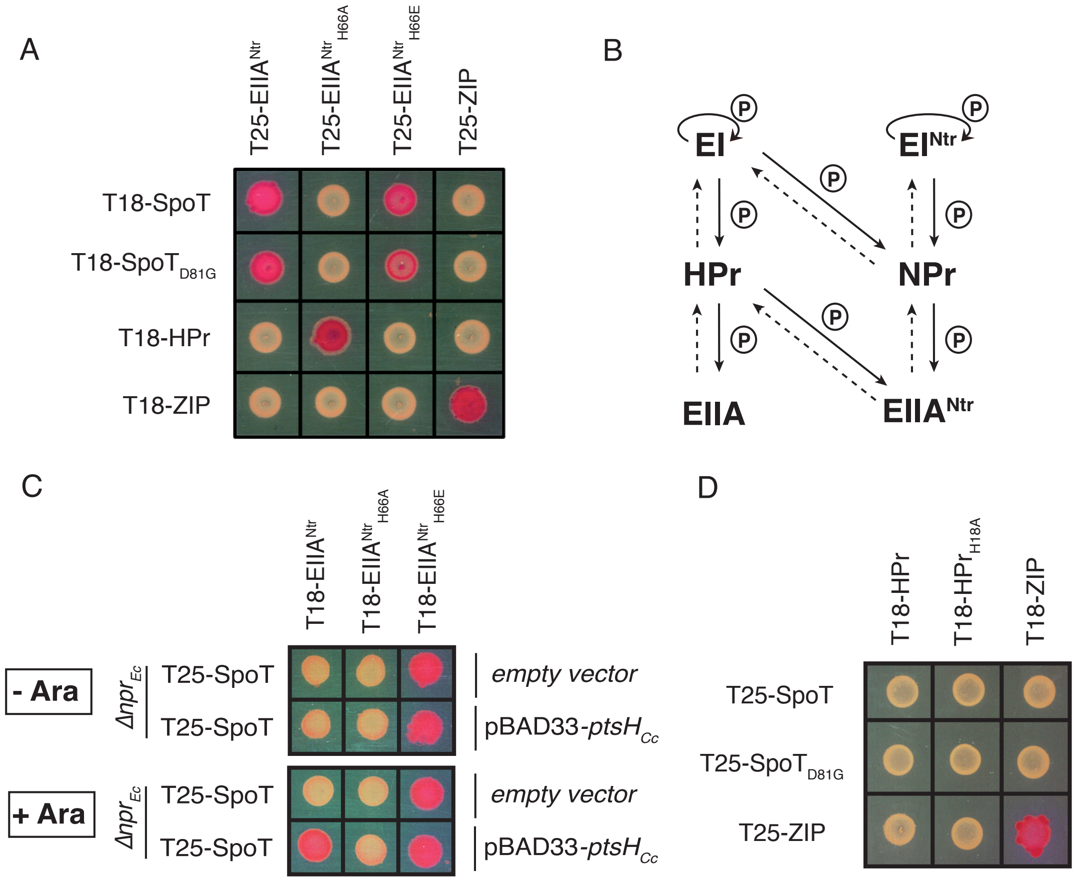

Supplementary Figure 9. EIIA^{Ntr}~P physically interacts with SpoT.

(**A**) EIIA^{Ntr}~P interacts with SpoT (or SpoT$_{D81G}$) in a BTH assay. MG1655 *cyaA::frt* (RH785) strain coexpressing T18- or T25- fused to *ptsN*, *ptsN$_{H66A}$, ptsN$_{H66E}$, spoT*, *spoT$_{D81G}$*, *ptsH, ptsH$_{H18A}$* or *ZIP* were spotted on MacConkey Agar Base plates supplemented with 1% maltose and 1 mM IPTG. Plates were incubated for 3-4 days at 30 °C. The red color indicates positive interactions.

(**B**) Schematic representation of the PTS and PTS^{Ntr} pathways in *E. coli*. NPr and EIIA^{Ntr} are can also be phosphorylated by the PTS system.

(**C**) *Caulobacter* HPr (HPr$_{Cc}$) restores the interaction between EIIA^{Ntr} and SpoT in a Δ*npr* background. MG1655 *cyaA::frt* Δ*npr* (RH2122) strain harbouring pBAD33 or pBAD33-*ptsH* and coexpressing T18- or T25- fused to *ptsN*, *ptsN$_{H66A}$, ptsN$_{H66E}$* or *spoT* were spotted on MacConkey Agar Base plates supplemented with 1% maltose,1 mM IPTG and with or without 0.05% arabinose. Plates were incubated for 3-4 days at 30 °C. The red color indicates positive interactions.

(**D**) Neither HPr nor HPr_{H18A} interacts with SpoT (or $SpoT_{D81G}$) in a BTH assay. MG1655 *cyaA::frt* (RH785) strain coexpressing T18- or T25- fused to *ptsH, $ptsH_{H18A}$,* *spoT*, *$spoT_{D81G}$*, or *ZIP* were spotted on MacConkey Agar Base plates supplemented with 1% maltose and 1 mM IPTG. Plates were incubated for 3-4 days at 30 °C. The red color indicates positive interactions.

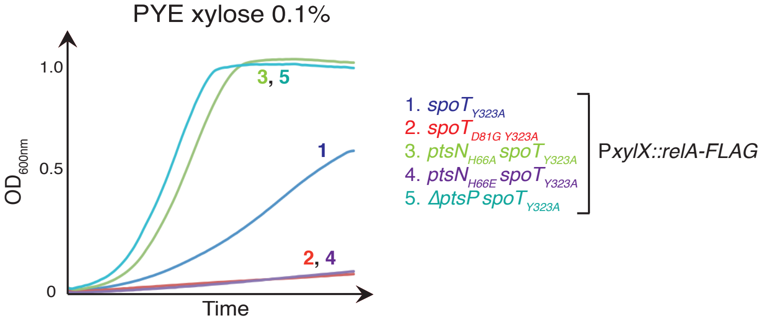

Supplementary Figure 10. The hydrolase activity of SpoT is required for growth upon artificial accumulation of (p)ppGpp.

Growth of $spoT_{Y323A}$, $spoT_{D81G\ Y323A}$, $ptsN_{H66A}$ $spoT_{Y323A}$, $ptsN_{H66E}$ $spoT_{Y323A}$ and Δ*ptsP* $spoT_{Y323A}$ containing the pXTCYC-4-*relA-FLAG* vector (P*xylX::relA-FLAG*) in PYE medium supplemented with 0.1% xylose.

Supplementary Table 1. Oligonucleotides used in this study.

Inserted restriction sites are indicated in capital letter

Name	Sequence
147	ttttAAGCTTatggtcgagaccgcctccat
148	cttagtcGAATTCaccgtcgacgacgtgttcca
149	cttagtcGAATTCctggagcagaacgaagccag
150	cttagtcGGATCCcaaggtcatcgccgcccagt
190	tcctggctcagtcgggttgc
191	cgggcctgatcgaaagcgtc
250	ttttAAGCTTgggtttttcgcgcgacat
251	cttagtcGAATTCgacgaccgctatgatcagtt
252	cttagtcGAATTCgaaaccggctcggccgctct
253	cttagtcGGATCCacgttgctcgcaggggtca
254	cgcctgtttcggtatagacg
255	acattgaCATATGgatgacggcttcgatcttct
256	acattgaCATATGatccgcatccgaaccggaga
257	cttagtcGGATCCcggcagttcggtcgaatcga
258	ttttAAGCTTatcgtaggcgtcaaatgaagc
259	cttagtcGAATTCcaggatgtccttggcggtgc
260	gatgcgcctgcaactgcacc
261	cttagtcGGATCCacgcggccgttcgaggattc
262	cttagtcGGATCCtgattgtcgccgtctcg
263	cttagtcGAATTCaatcacggcgacgatcatct
264	cttagtcGAATTCatcgccggctgacccaaaca
265	ttttAAGCTTcgtcgatgatcaccgccttc
311	tgcccaatgcgctgttcgac
312	tcgaggtgccgggtatcatc

313	cacataggccgcgttccagg
314	cgcgaaggtcgactggaagg
315	catccgcttcatcagcgagc
316	aggtcttggcagctgtcgac
317	caaaaccggtgtgtcgtcgc
318	cagatcgaagtcggcgttgc
341	cttagtcGGATCCagggcgatcatcggattgc
342	cttagtcGAATTCcgcttcatacgcccgctcag
343	cttagtcGAATTCgcggcctatgaggcggtcgt
344	gtccctgcgcttgaagcgct
345	cttagtcAAGCTTcgtacgctgccaatgacgac
346	cttagtcGAATTCcaggatctggtcgcgcttgg
347	cttagtcGAATTCgagcgggagttcctgctgct
348	cttagtcGGATCCagatagtccgcgtcctcag
349	cttagtcAAGCTTcgtcttcgtcttgggcaagg
350	cttagtcGAATTCgatgtcggctacagcgctca
351	cttagtcGAATTCgactggtacctgcgcaacgc
352	cttagtcGGATCCagaacttccggccgggatcg
383	caccgtcatggtctttgtagtc
435	gctcctggtcgatgcggatg
436	gatcgttcccgtcggagacc
437	cggaggctgtgtcatgaggc
438	agccatagcgatcggccatc
439	tcatcgacgagacccacacg
440	accttgctcttgcggtagcc
648	cgacgaaaccgatcggatcc
649	cttagtcGAATTCgaggacgagacggatagagc
650	cttagtcGAATTCttgggcctcaaccgcaacac
651	cttagtcAAGCTTcgacgtaggaattggcgacc

652	cttagtcAAGCTTgagttctcagcgttcgctcg
653	cttagtcGAATTCgccttcatcctccaggatgc
654	cttagtcGAATTCctgcaccgtaagctgaagtcc
655	cttagtcGGATCCgacaaaccacgcctcgatcg
727	tctacttcggccctgaagcc
728	gcagttcggtcgaatcgaacg
729	ttttCATATGagcaccgccaaggacatcc
730	ccGGTACCcttggcgtttgtcctaagcc
762	ctgttcgccggcgacaagta
763	cttcagctcgatgtagctgtcg
779	cgcgcaactgttgaagacgg
780	cggccgaaaatccgttgtcc
781	gactttcgatcgctgcaccg
782	acagcgtatagcgggtcacg
802	gactaagAAGCTTcagatcgtcgagacccatgg
803	gactaagGGTACCcacgagcccgatcatgcctt
804	gactaagGGTACCgtgctcgccggagaaaagtg
805	gactaagGGATCctacgaactggtggcgatcg
806	gactaagAAGCTTctaacgggcgtatcaagacc
807	gactaagGGTACCcgttcttgaagccatgcccg
808	gactaagGGTACCgaggagcggtaacaccctcc
809	gactaagGGATCCggcctatgggcccaactacg
814	agaccctggacctcgtcacc
815	atgcgcgtcaggggaagtcg
825	caccaagacgagaacatccaagc
826	aggtcaccaagtggctgaag
877	ttcgtagtcgttccaaagccgc
878	ccagcatctggtcgatctgg
885	ccgcgatttcctgactgacc

894	AAGCTTacacctcatcagcacgctgc
895	GAATTCagacataggcgcggttcagc
896	GAATTCtcttcgacaccgacatcgacg
897	GGATCCcgctgtcgaaagcttcgagg
898	gatggtcgaccgcgataacg
899	gcgcgagatccatgaagagc
900	gatattgctgaagagcttggcggcgaa
905	AAGCTTgcggttgcatcgagggatgg
906	GGTACCgatgccggatgccgccatga
907	GGTACCcgcaagctctacgtcgccgt
908	GGATCcgttcttgaagcgggccacg
923	cgcctaagcggaacaacGCG
944	AAGCTTccctggccaagatcgatacg
945	GGTACCgcaaccatggaaagcgcctg
946	GGTACCcgtcctgctgaccgacgaac
947	GGATccctctgacggccatgtgga
961	gactacaaagaccatgacggtg
963	Gtcgtggtcagcaaggaagg
964	Gtcggccatgaccaccttgg
972	Gagatggtcagctccagcgag
973	cgaacaggcgataggtctcc
1078	ccatacgatgttccagattacgc
1126	cgtcggtggattatcgtcgg
1175	TCTAGAgttgtcgtctgtggcccccgtcgctg
1176	GAATTcctgaaggagagggtcctcg
1177	TCTAGAggtgacgggcatggcttcaagaacg
1178	GAATTCtaccgctcctcgtcgaaccgga
1181	TCTAGAggtgtcatggtctggcgccgtcgg
1182	GAATTCgaagaaggtcagaagggcgtc

1262 ATGCTCACTCGCCTGCGCGAAATAGTCGAAAAGGTAGCCgtgtaggctggagctgcttc

1263 CTATAACCCTCCGCGAATCAGCCCGCCCATGCCGCGACGcatatgaatatcctcctta

1273 gactcaaggtaccgttgtga

1274 ttcttgtcgtcggaaaccag

1275 aacgcatctgcttatcgacg

1276 ctccggaaaatgcagatagc

1310 tccagtcacgccatcgtacg

1311 ttgggcatcttccggatgcg

1349 cctaagtaactaaTCTAGAaggagggagtaatgacgggcatggcttcaagaacg

1350 ggatccccgggtaCTGCAGaggaggcggagggtgttacc

Supplementary Table 2. Plasmids used in this study.

Name	description	Reference
	pNPTS138	M. R. Alley, Imperial College London (UK), unpublished
	pRVMCS-5	[2]
	pNPTS138-*mipZ::mipZ-mcfp*	[3]
	pNPTS138-*HA-spoT*	[4]
	pKT25	[5]
	pUT18C	[5]
	pKT25-*zip*	[5]
	pUT18C-*zip*	[5]
	pCP20	[6]
	pBAD33	[7]
	pXTCYC-4-*relA-FLAG*	[8]
pHR322	pNPTS138-Δ*glnD*	This study
pHR366	pNPTS138-Δ*glnK*	This study
pHR367	pNPTS138-Δ*glnB*	This study
pHR368	pNPTS138-Δ*glnA*	This study
pHR369	pNPTS138-Δ*glnC*	This study
pHR389	pNPTS138-Δ*glnE*	This study
pHR390	pNPTS138-Δ$glnA_2$	This study
pHR391	pNPTS138-Δ$glnA_3$	This study
pHR523	pNPTS138-Δ*ptsN*	This study
pHR533	pRVMCS-5-*glnA*	This study
pHR542	pNPTS138-Δ*ntrC*	This study
pHR543	pNPTS138-Δ*ntrX*	This study
pHR545	pNPTS138-$glnA_{R360A}$	This study
pHR589	pNPTS138-Δ*ptsM*	This study
pHR590	pNPTS138-Δ*ptsH*	This study

pHR594	pNPTS138-*ptsH*$_{H18A}$	This study
pHR628	pNPTS138-*ptsP*$_{L83Q}$	This study
pHR634	pNPTS138-*spoT*$_{D81G}$	This study
pHR638	pNPTS138-Δ*spoT*	This study
pHR639	pNPTS138-Δ*ptsP*	This study
pHR645	pNPTS138-*spoT*$_{Y323A}$	This study
pHR689	pUT18C-*spoT*	This study
pHR690	pUT18C-*ptsH*	This study
pHR692	pUT18C-*spoT*$_{D81G}$	This study
pHR693	pKT25-*ptsN*	This study
pHR694	pKT25-*ptsN*$_{H66A}$	This study
pHR695	pKT25-*ptsN*$_{H66E}$	This study
pHR699	pXMCS-2-3FLAG-*ptsN*	This study
pHR704	pUT18C-*ptsN*	This study
pHR705	pUT18C-*ptsN*$_{H66E}$	This study
pHR706	pUT18C-*ptsN*$_{H66A}$	This study
pHR711	pNPTS138-*ptsN*$_{H66E}$	This study
pHR712	pNPTS138-*ptsN*$_{H66A}$	This study
pHR764	pKT25-*spoT*	This study
pHR765	pKT25-*spoT*$_{D81G}$	This study
pHR813	pBAD33-*ptsH*	This study
pHR815	pKT25-*ptsH*	This study
pHR816	pKT25-*ptsH*$_{H18A}$	This study

Supplementary Table 3. Strains used in this study.

Name	Description and relevant genotype	Reference
RH10	S17-1 ((F- λ- *22nd Athi pro recA hsdr2* (r-m+) RP4-2-Tet ::Mu-Km ::Tn7)	[9]
RH319	MT607(*pro*-82 *thi*-I *hsdR17* (r-m+) *supE44 recA56*)	[10]
RH392	MG1655 mini λ^{Tet}	L. Van Melderen, ULB (Belgium)
RH783	Top10 (*φ80lacZΔM15 araD139* Δ(*ara-leu*)7697 *galE15 galK16* Δ(*lac*)X74 *rpsL*(StrR) *nupG recA1 endA1 mcrA* Δ (*mrr-hsdRMS-mcrBC*)	Life Technologies
RH785	MG1655 *cya ::frt*	[11]
RH2084	MG1655 Δ*ptsP::cat*	This study
RH2117	MG1655 *cya ::frt* Δ*ptsP*	This study
RH2122	MG1655 *cya ::frt* Δ*npr*	This study
RH2124	MG1655 *cya ::frt* Δ*ptsP* Δ*ptsI*	This study
RH50	NA1000	[12]
RH507	NA1000 *stpX::stpX-gfp*	[13]
RH522	NA1000 *mipZ::mipZ-mcfp*	[3]
RH577	NA1000 Δ*glnD*	This study
RH737	NA1000 Δ*glnD stpX::stpX-gfp*	This study
RH770	NA1000 Δ*glnK*	This study
RH771	NA1000 Δ*glnB*	This study
RH772	NA1000 Δ*glnA*	This study
RH773	NA1000 Δ*glnC*	This study
RH778	NA1000 Δ*glnK* Δ*glnC*	This study
RH793	NA1000 Δ*glnD mipZ::mipZ-mcfp*	This study
RH874	NA1000 Δ*glnE*	This study
RH875	NA1000 Δ*glnD* Δ*glnE*	This study
RH876	NA1000 Δ$glnA_2$	This study

RH877	NA1000 Δ*glnA*$_{3}$	This study
RH878	NA1000 Δ*glnA*$_{2}$ Δ*glnA*$_{3}$	This study
RH879	NA1000 Δ*glnA*$_{2}$ Δ*glnA*$_{3}$ Δ*glnA*	This study
RH970	NA1000 Δ*glnA*$_{2}$ Δ*glnA*	This study
RH971	NA1000 Δ*glnA*$_{3}$ Δ*glnA*	This study
RH1458	NA1000 Δ*ntrC*	This study
RH1459	NA1000 Δ*ntrX*	This study
RH1621	NA1000 Δ*ptsH*	This study
RH1622	NA1000 Δ*ptsM*	This study
RH1632	NA1000 *glnA*$_{R360A}$	This study
RH1702	NA1000 Δ*ptsH* Δ*ptsM*	This study
RH1727	NA1000 Δ*ptsP spoT*$_{D81G}$	This study
RH1728	NA1000 Δ*spoT ptsP*$_{L83Q}$	This study
RH1748	NA1000 *ptsP*$_{L83Q}$	This study
RH1752	NA1000 *spoT*$_{D81G}$	This study
RH1753	NA1000 Δ*glnD spoT*$_{D81G}$	This study
RH1755	NA1000 Δ*spoT*	This study
RH1756	NA1000 Δ*glnD* Δ*spoT*	This study
RH1758	NA1000 Δ*ptsP*	This study
RH1782	NA1000 Δ*ptsM ptsH*$_{H18A}$	This study
RH1819	NA1000 Δ*ptsN*	This study
RH1829	NA1000 Δ*ptsM* Δ*ptsN*	This study
RH1844	NA1000 *spoT*$_{Y323A}$	This study
RH1888	NA1000 *3FLAG-ptsN*	This study
RH1940	NA1000 Δ*glnD* Δ*ptsP*	This study
RH1941	NA1000 *ptsP*$_{L83Q}$Δ*glnD*	This study
RH1999	NA1000 *spoT*$_{D81G}$ Δ*ptsN*	This study
RH2013	NA1000 *spoT*$_{D81G}$ Δ*ptsH*	This study

RH2014	NA1000 $spoT_{D81G}$ Δ*ptsN* Δ*ptsP*	This study
RH2015	NA1000 $spoT_{D81G}$ Δ*ptsN* Δ*ptsH*	This study
RH2016	NA1000 Δ*ptsP* $ptsN_{H66E}$	This study
RH2017	NA1000 $ptsN_{H66E}$	This study
RH2018	NA1000 $ptsP_{L83Q}$ $ptsN_{H66A}$	This study
RH2191	NA1000 pXMCS-2-*3FLAG-ptsN*	This study
RH2192	NA1000 Δ*ptsP* pXMCS-2-*3FLAG-ptsN*	This study
RH2193	NA1000 $spoT_{D81G\ Y323A}$	This study
RH2194	NA1000 $ptsN_{H66A}$ $spoT_{Y323A}$	This study
RH2195	NA1000 $ptsN_{H66E}$ $spoT_{Y323A}$	This study
RH2196	NA1000 Δ*ptsP* $spoT_{Y323A}$	This study

Supplementary Methods

Plasmids construction

pHR322 (pNPTS138-Δ*glnD*)

Upstream and downstream regions of *C. crescentus CCNA_00013* were amplified from NA1000 gDNA by PCR respectively with primers 147/148 and 149/150 and cloned into pSK. The pSK-147/148 and pSK-149/150 recombinant plasmids were then digested respectively with *Hind* III/*Eco* RI and *Eco* RI/*Bam* HI; and ligated into the pNPTS138 vector cut with *Hind* III and *Bam* HI.

pHR366 (pNPTS138-Δ*glnK*)

Upstream and downstream regions of *C. crescentus CCNA_01400* were amplified from NA1000 gDNA by PCR respectively with primers 250/251 and 252/253 and cloned into pSK. The pSK-250/251 and pSK-252/253 recombinant plasmids were then digested respectively with *Hind* III/*Eco* RI and *Eco* RI/*Bam* HI; and ligated into the pNPTS138 vector cut with *Hind* III and *Bam* HI.

pHR367 (pNPTS138-Δ*glnB*)

Upstream and downstream regions of *C. crescentus CCNA_02046* were amplified from NA1000 gDNA by PCR respectively with primers 254/255 and 256/257 and cloned into pSK. The pSK-254/255 and pSK-256/257 recombinant plasmids were then digested respectively with *Hind* III/*Nde* I and *Nde* I/*Bam* HI; and ligated into the pNPTS138 vector cut with *Hind* III and *Bam* HI.

pHR368 (pNPTS138-Δ*glnA*)

Upstream and downstream regions of *C. crescentus CCNA_02047* were amplified from NA1000 gDNA by PCR respectively with primers 258/259 and 260/261 and cloned into pSK. The pSK-258/259 and pSK-260/261 recombinant plasmids were then digested respectively with *Hind* III/*Eco* RI and *Eco* RI/*Bam* HI; and ligated into the pNPTS138 vector cut with *Hind* III and *Bam* HI.

pHR369 (pNPTS138-Δ*glnC*)

Upstream and downstream regions of *C. crescentus CCNA_00555* were amplified from NA1000 gDNA by PCR respectively with primers 262/263 and 264/265 and cloned into pSK. The pSK-262/263 and pSK-264/265 recombinant plasmids were then digested respectively with *Bam* HI/*Eco* RI and *Eco* RI/*Hind* III; and ligated into the pNPTS138 vector cut with *Hind* III and *Bam* HI.

pHR389 (pNPTS138-Δ*glnE*)

Upstream and downstream regions of *C. crescentus CCNA_02839* were amplified from NA1000 gDNA by PCR respectively with primers 341/342 and 343/344 and cloned into pSK. The pSK-341/342 and pSK-343/344 recombinant plasmids were then digested respectively with *Bam* HI/*Eco* RI and *Eco* RI/*Hind* III; and ligated into the pNPTS138 vector cut with *Hind* III and *Bam* HI.

pHR390 (pNPTS138-Δ$glnA_2$)

Upstream and downstream regions of *C. crescentus CCNA_03230* were amplified from NA1000 gDNA by PCR respectively with primers 345/346 and 347/348 and cloned into pSK. The pSK-345/346 and pSK-347/348 recombinant plasmids were then digested respectively with *Hind* III/*Eco* RI and *Eco* RI/*Bam* HI; and ligated into the pNPTS138 vector cut with *Hind* III and *Bam* HI.

pHR391 (pNPTS138-Δ$glnA_3$)

Upstream and downstream regions of *C. crescentus CCNA_03240* were amplified from NA1000 gDNA by PCR respectively with primers 349/350 and 351/352 and cloned into pSK. The pSK-349/350 and pSK-351/352 recombinant plasmids were then digested respectively with *Hind* III/*Eco* RI and *Eco* RI/*Bam* HI; and ligated into the pNPTS138 vector cut with *Hind* III and *Bam* HI.

pHR523 (pNPTS138-Δ*ptsN*)

Upstream and downstream regions of *C. crescentus CCNA_03710* were amplified from NA1000 gDNA by PCR respectively with primers 944/945 and 946/947 and cloned into pSK. The pSK-944/945 and pSK-946/947 recombinant plasmids were then digested respectively with *Hind* III/*Asp* 718 and *Asp* 718/*Bam* HI; and ligated into the pNPTS138 vector cut with *Hind* III and *Bam* HI.

pHR533 (pRVMCS-5-*glnA*)

C. crescentus CCNA_02047 was amplified from NA1000 gDNA by PCR with primers 729 and 730 and cloned into pSK. The pSK-729/730 recombinant plasmid was then digested with *Nde* I *and Asp* 718 and ligated into the pRVMCS-5 vector cut with the same restriction enzymes.

pHR542 (pNPTS138-Δ*ntrC*)

Upstream and downstream regions of *C. crescentus CCNA_01815* were amplified from NA1000 gDNA by PCR respectively with primers 648/649 and 650/651 and

cloned into pSK. The pSK-648/649 and pSK-650/651 recombinant plasmids were then digested respectively with *Hind* III/*Eco* RI and *Eco* RI/*Bam* HI; and ligated into the pNPTS138 vector cut with *Hind* III and *Bam* HI.

pHR543 (pNPTS138-Δ*ntrX*)

Upstream and downstream regions of *C. crescentus CCNA_01817* were amplified from NA1000 gDNA by PCR respectively with primers 652/653 and 654/655 and cloned into pSK. The pSK-652/653 and pSK-654/655 recombinant plasmids were then digested respectively with *Hind* III/*Eco* RI and *Eco* RI/*Bam* HI; and ligated into the pNPTS138 vector cut with *Hind* III and *Bam* HI.

pHR545 (pNPTS138-$glnA_{R360A}$)

DNA fragment of *C. crescentus CCNA_02047* encompassing catalytic site mutation R360A was synthesized as a gBlock (ITD), amplified by PCR with primers 762/763 and cloned into pNPTS138 cut with *Eco* RV.

pHR589 (pNPTS138-Δ*ptsM*)

Upstream and downstream regions of *C. crescentus CCNA_00240* were amplified from NA1000 gDNA by PCR respectively with primers 802/803 and 804/805 and cloned into pSK. The pSK-802/803 and pSK-804/805 recombinant plasmids were then digested respectively with *Hind* III/*Asp* 718 and *Asp* 718/*Bam* HI; and ligated into the pNPTS138 vector cut with *Hind* III and *Bam* HI.

pHR590 (pNPTS138-Δ*ptsH*)

Upstream and downstream regions of *C. crescentus CCNA_00241* were amplified from NA1000 gDNA by PCR respectively with primers 806/807 and 808/809 and cloned into pSK. The pSK-806/807 and pSK-808/809 recombinant plasmids were then digested respectively with *Hind* III/*Asp* 718 and *Asp* 718/*Bam* HI; and ligated into the pNPTS138 vector cut with *Hind* III and *Bam* HI.

pHR594 (pNPTS138-$ptsH_{H18A}$)

DNA fragment of *C. crescentus CCNA_00241* encompassing the non-phosphorylatable mutation R360A was synthesized as a gBlock (ITD), amplified by PCR with primers 825/826 and cloned into pNPTS138 cut with *Eco* RV.

pHR628 (pNPTS138-$ptsP_{L83Q}$)

C. crescentus CCNA_00892 with the mutation L83Q was amplified from a gain-of-function mutant isolated on swarm agar plates supplemented with 9 mM Glutamine by PCR with primers 877 and 878 and cloned into pNPTS138 cut with *Eco* RV.

pHR634 (pNPTS138-*spoT*$_{D81G}$)

C. crescentus CCNA_01622 with the mutation D81G was amplified from a gain-of-function mutant isolated on swarm agar plates supplemented with 9 mM Glutamine by PCR with primers 894 and 885 and cloned into pNPTS138 cut with *Eco* RV.

pHR638 (pNPTS138-Δ*spoT*)

Upstream and downstream regions of *C. crescentus CCNA_01622* were amplified from NA1000 gDNA by PCR respectively with primers 894/895 and 896/897 and cloned into pSK. The pSK-894/895 and pSK-896/897 recombinant plasmids were then digested respectively with *Hind* III/*Eco* RI and *Eco* RI/*Bam* HI; and ligated into the pNPTS138 vector cut with *Hind* III and *Bam* HI.

pHR639 (pNPTS138-Δ*ptsP*)

Upstream and downstream regions of *C. crescentus CCNA_00892* were amplified from NA1000 gDNA by PCR respectively with primers 905/906 and 907/908 and cloned into pSK. The pSK-905/906 and pSK-907/908 plasmids were then digested respectively with *Hind* III/*Asp* 718 and *Asp* 718/*Bam* HI; and ligated into the pNPTS138 vector cut with *Hind* III and *Bam* HI.

pHR645 (pNPTS138-*spoT*$_{Y323A}$)

DNA fragment of *C. crescentus CCNA_01622* encompassing catalytic site mutation Y323A was synthesized as a gBlock (ITD), amplified by PCR with primers 932/933 and cloned into pNPTS138 cut with *Eco* RV.

pHR689 (pUT18C-*spoT*)

C. crescentus CCNA_01622 was amplified from NA1000 gDNA by PCR with primers 1175 and 1176 and cloned into pSK. The pSK-1175/1176 recombinant plasmid was then digested with *Xba* I and *Eco* RI and ligated into the pUT18C vector cut with the same restriction enzymes.

pHR690 (pUT18C-*ptsH*)

C. crescentus CCNA_00241 was amplified from NA1000 gDNA by PCR with primers 1177 and 1178 and cloned into pSK. The pSK-1177/1178 recombinant plasmid was

then digested with *Xba* I *and Eco* RI and ligated into the pUT18C vector cut with the same restriction enzymes.

pHR692 (pUT18C-*spoT*$_{D81G}$)

C. crescentus CCNA_01622 with the mutation D81G was amplified from a gain-of-function mutant isolated on swarm agar plates supplemented with 9 mM Glutamine by PCR with primers 1175 and 1176 and cloned into pSK. The pSK-1175/1176 recombinant plasmid was then digested with *Xba* I and *Eco* RI and ligated into the pUT18C vector cut with the same restriction enzymes.

pHR693 (pKT25-*ptsN*)

C. crescentus CCNA_03710 was amplified from NA1000 gDNA by PCR with primers 1181 and 1182 and cloned into pSK. The pSK-1181/1182 recombinant plasmid was then digested with *Xba* I *and Eco* RI and ligated into the pKT25 vector cut with the same restriction enzymes.

pHR694 (pKT25-*ptsN*$_{H66A}$)

DNA fragment of *C. crescentus CCNA_03710* encompassing mutations H66A was synthesized as a gBlock (IDT) and cloned into pSK. The pSK- *ptsN*$_{H66A}$ recombinant plasmid was then digested with *Xba* I and *Eco* RI and ligated into the pKT25 vector cut with the same restriction enzymes.

pHR695 (pKT25-*ptsN*$_{H66E}$)

C. crescentus CCNA_03710 was amplified from RH2017 by PCR with primers 1181 and 1182 and cloned into pSK. The pSK- *ptsN*$_{H66E}$ recombinant plasmid was then digested with *Xba* I and *Eco* RI and ligated into the pKT25 vector cut with the same restriction enzymes.

pHR704 (pUT18C-*ptsN*)

C. crescentus CCNA_03710 was amplified from NA1000 gDNA by PCR with primers 1181 and 1182 and cloned into pSK. The pSK-1181/1182 recombinant plasmid was then digested with *Xba* I and *Eco* RI and ligated into the pUT18C vector cut with the same restriction enzymes.

pHR699 (pXMCS-2-*3FLAG-ptsN*)

3FLAG-ptsN was amplified from RH1888 (NA1000 *3FLAG-ptsN*) by PCR with primers 1173 and 1174 and cloned into pSK. The pSK-*3FLAG-ptsN* was then

digested with *NdeI* and *Eco* RI and ligated into the pXMCS-2 cut with the same restriction enzymes.

pHR705 (pUT18C-*ptsN*$_{H66E}$)

DNA fragment of *C. crescentus CCNA_03710* encompassing mutations H66E was synthesized as a gBlock (IDT) and cloned into pSK. The pSK- *ptsN*$_{H66E}$ recombinant plasmid was then digested with *Xba* I and *Eco* RI and ligated into the pUT18C vector cut with the same restriction enzymes.

pHR706 (pUT18C-*ptsN*$_{H66A}$)

DNA fragment of *C. crescentus CCNA_03710* encompassing mutations H66A was synthesized as a gBlock (IDT) and cloned into pSK. The pSK- *ptsN*$_{H66A}$ recombinant plasmid was then digested with *Xba* I and *Eco* RI and ligated into the pUT18C vector cut with the same restriction enzymes.

pHR711 (pNPTS138-*ptsN*$_{H66E}$)

DNA fragment of *C. crescentus CCNA_03710* encompassing mutations H66E was synthesized as a gBlock (IDT) and cloned into pNPTS138 cut with *Eco* RV.

pHR712 (pNPTS138-*ptsN*$_{H66A}$)

DNA fragment of *C. crescentus CCNA_03710* encompassing mutations H66A was synthesized as a gBlock (IDT) and cloned into pNPTS138 cut with *Eco* RV.

pHR764 (pKT25-*spoT*)

C. crescentus CCNA_01622 was amplified from NA1000 gDNA by PCR with primers 1175 and 1176 and cloned into pSK. The pSK-1175/1176 recombinant plasmid was then digested with *Xba* I and *Eco* RI and ligated into the pKT25 vector cut with the same restriction enzymes.

pHR765 (pKT25-*spoT*$_{D81G}$)

C. crescentus CCNA_01622 with the mutation D81G was amplified from a gain-of-function mutant isolated on swarm agar plates supplemented with 9 mM Glutamine by PCR with primers 1175 and 1176 and cloned into pSK. The pSK-1175/1176 recombinant plasmid was then digested with *Xba* I and *Eco* RI and ligated into the pKT25 vector cut with the same restriction enzymes.

pHR813 (pBAD33-*ptsH*)

C. crescentus CCNA_00241 was amplified from NA1000 gDNA by PCR with primers 1349 and 1350 and cloned into pSK. The pSK-1349/1350 recombinant plasmid was

then digested with *Xba* I and *Pst* I and ligated into the pBAD33 vector cut with the same restriction enzymes.

pHR815 (pKT25-*ptsH*)

C. crescentus CCNA_00241 was amplified from NA1000 gDNA by PCR with primers 1177 and 1178 and cloned into pSK. The pSK-1177/1178 recombinant plasmid was then digested with *Xba* I and *Eco* RI and ligated into the pKT25 vector cut with the same restriction enzymes.

pHR816 (pKT25-$ptsH_{H18A}$)

C. crescentus CCNA_00241 with the mutation H18A was amplified by PCR with primers 1177 and 1178 and cloned into pSK. The pSK-1177/1178 recombinant plasmid was then digested with *Xba* I and *Eco* RI and ligated into the pKT25 vector cut with the same restriction enzymes.

Description of Strains

RH577 (NA1000 Δ*glnD*)

Biparental mating between NA1000 and RH528 (S17-1-pNPTS138-Δ*glnD*) was selected on PYE Nal Kan, streaked on PYE Kan, cultivated o/n in PYE and plated on PYE 3% Suc. Suc^R candidates were streaked on PYE Kan and PYE. Kan^S colonies were screened by PCR with primers 190/191.

RH737 (NA1000 Δ*glnD stpX::stpX-gfp*)

A CR30 lysate made on RH507 (NA1000 *stpX::stpX-gfp*) was transduced into RH577 (NA1000 Δ*glnD*). Transductants were selected on PYE Kan.

RH770 (NA1000 Δ*glnK*)

Biparental mating between NA1000 and RH766 (S17-1-pNPTS138-Δ*glnK*) was selected on PYE Nal Kan, streaked on PYE Kan, cultivated o/n in PYE and plated on PYE 3% Suc. Suc^R candidates were streaked on PYE Kan and PYE. Kan^S colonies were screened by PCR with primers 313/314.

RH771 (NA1000 Δ*glnB*)

Biparental mating between NA1000 and RH768 (S17-1-pNPTS138-Δ*glnA*) was selected on PYE Nal Kan, streaked on PYE Kan, cultivated o/n in PYE and plated on PYE 3% Suc. Suc^R candidates were streaked on PYE Kan and PYE. Kan^S colonies were screened by PCR with primers 315/316.

RH772 (NA1000 Δ*glnA*)

Biparental mating between NA1000 and RH767 (S17-1-pNPTS138-Δ*glnB*) was selected on PYE Nal Kan, streaked on PYE Kan, cultivated o/n in PYE and plated on PYE 3% Suc. Suc^R candidates were streaked on PYE Kan and PYE. Kan^S colonies were screened by PCR with primers 317/318.

RH773 (NA1000 Δ*glnC*)

Biparental mating between NA1000 and RH769 (S17-1-pNPTS138-Δ*glnC*) was selected on PYE Nal Kan, streaked on PYE Kan, cultivated o/n in PYE and plated on PYE 3% Suc. Suc^R candidates were streaked on PYE Kan and PYE. Kan^S colonies were screened by PCR with primers 311/312.

RH778 (NA1000 Δ*glnK* Δ*glnC*)

Biparental mating between RH770 (NA1000 Δ*glnK*) and RH769 (S17-1-pNPTS138-Δ*glnC*) was selected on PYE Nal Kan, streaked on PYE Kan, cultivated o/n in PYE and plated on PYE 3% Suc. Suc^R candidates were streaked on PYE Kan and PYE. Kan^S colonies were screened by PCR with primers 311/312.

RH793 (NA1000 Δ*glnD mipZ::mipZ-mcfp*)

Biparental mating between RH577 (NA1000 Δ*glnD*) and S17-1-pNPTS138-*mipZ-mcfp* was selected on PYE Nal Kan, streaked on PYE Kan, cultivated o/n in PYE and plated on PYE 3% Suc. Suc^R candidates were streaked on PYE Kan and PYE. Kan^S colonies were screened by fluorescence microscopy.

RH874 (NA1000 Δ*glnE*)

Biparental mating between NA1000 and RH871 (S17-1-pNPTS138-Δ*glnE*) was selected on PYE Nal Kan, streaked on PYE Kan, cultivated o/n in PYE and plated on PYE 3% Suc. Suc^R candidates were streaked on PYE Kan and PYE. Kan^S colonies were screened by PCR with primers 435/436.

RH875 (NA1000 Δ*glnD* Δ*glnE*)

Biparental mating between RH577 (NA1000 Δ*glnD*) and RH871 (S17-1-pNPTS138-Δ*glnE*) was selected on PYE Nal Kan, streaked on PYE Kan, cultivated o/n in PYE and plated on PYE 3% Suc. Suc^R candidates were streaked on PYE Kan and PYE. Kan^S colonies were screened by PCR with primers 435/436.

RH876 (NA1000 Δ$glnA_2$)

Biparental mating between NA1000 and RH872 (S17-1-pNPTS138-Δ$glnA_2$) was

selected on PYE Nal Kan, streaked on PYE Kan, cultivated o/n in PYE and plated on PYE 3% Suc. Suc^R candidates were streaked on PYE Kan and PYE. Kan^S colonies were screened by PCR with primers 437/438.

RH877 (NA1000 Δ*glnA$_3$*)

Biparental mating between NA1000 and RH873 (S17-1-pNPTS138-Δ*glnA$_3$*) selected on PYE Nal Kan, streaked on PYE Kan, cultivated o/n in PYE and plated on PYE 3% Suc. Suc^R candidates were streaked on PYE Kan and PYE. Kan^S colonies were screened by PCR with primers 439/440.

RH878 (NA1000 Δ*glnA$_2$* Δ*glnA$_3$*)

Biparental mating between RH876 (NA1000 Δ*glnA$_2$*) and RH873 (S17-1-pNPTS138-Δ*glnA$_3$*) was selected on PYE Nal Kan, streaked on PYE Kan, cultivated o/n in PYE and plated on PYE 3% Suc. Suc^R candidates were streaked on PYE Kan and PYE. Kan^S colonies were screened by PCR with primers 439/440.

RH879 (NA1000 Δ*glnA$_2$* Δ*glnA$_3$* Δ*glnA*)

Biparental mating between RH878 (NA1000 Δ*glnA$_2$* Δ*glnA$_3$*) and RH767 (S17-1-pNPTS138-Δ*glnB*) was selected on PYE Nal Kan, streaked on PYE Kan, cultivated o/n in PYE and plated on PYE 3% Suc. Suc^R candidates were streaked on PYE Kan and PYE. Kan^S colonies were screened by PCR with primers 317/318.

RH970 (NA1000 Δ*glnA$_2$* Δ*glnA*)

Biparental mating between RH876 (NA1000 Δ*glnA$_2$*) and RH767 (S17-1-pNPTS138-Δ*glnB*) was selected on PYE Nal Kan, streaked on PYE Kan, cultivated o/n in PYE and plated on PYE 3% Suc. Suc^R candidates were streaked on PYE Kan and PYE. Kan^S colonies were screened by PCR with primers 317/318.

RH971 (NA1000 Δ*glnA$_3$* Δ*glnA*)

Biparental mating between RH877 (NA1000 Δ*glnA$_3$*) and RH767 (S17-1-pNPTS138-Δ*glnB*) was selected on PYE Nal Kan, streaked on PYE Kan, cultivated o/n in PYE and plated on PYE 3% Suc. Suc^R candidates were streaked on PYE Kan and PYE. Kan^S colonies were screened by PCR with primers 317/318.

RH1458 (NA1000 Δ*ntrC*)

Triparental mating between NA1000, RH319 (MT607-pRK600) and RH1403 (Top10-pNPTS138-Δ*ntrC*) was selected on PYE Nal Kan, streaked on PYE Kan, cultivated

o/n in PYE and plated on PYE 3% Suc. Suc^R candidates were streaked on PYE Kan and PYE. Kan^S colonies were screened by PCR with primers 779/780.

RH1459 (NA1000 Δ*ntrX*)

Triparental mating between NA1000, RH319 (MT607-pRK600) and RH1404 (Top10-pNPTS138-Δ*ntrX*) was selected on PYE Nal Kan, streaked on PYE Kan, cultivated o/n in PYE and plated on PYE 3% Suc. Suc^R candidates were streaked on PYE Kan and PYE. Kan^S colonies were screened by PCR with primers 781/782.

RH1621 (NA1000 Δ*ptsH*)

Triparental mating between NA1000, RH319 (MT607-pRK600) and RH1605 (Top10-pNPTS138-Δ*ptsH*) was selected on PYE Nal Kan, streaked on PYE Kan, cultivated o/n in PYE and plated on PYE 3% Suc. Suc^R candidates were streaked on PYE Kan and PYE. Kan^S colonies were screened by PCR with primers 814/815.

RH1622 (NA1000 Δ*ptsM*)

Triparental mating between NA1000, RH319 (MT607-pRK600) and RH1604 (Top10-pNPTS138-Δ*ptsM*) was selected on PYE Nal Kan, streaked on PYE Kan, cultivated o/n in PYE and plated on PYE 3% Suc. Suc^R candidates were streaked on PYE Kan and PYE. Kan^S colonies were screened by PCR with primers 814/815.

RH1632 (NA1000 $glnA_{R360A}$)

Triparental mating between NA1000, RH319 (MT607-pRK600) and RH1417 (Top10-pNPTS138-$glnA_{R360A}$) was selected on PYE Nal Kan, cultivated o/n in PYE and plated on PYE 3% Suc. Suc^R candidates were streaked on PYE 9.3 mM Gln Kan, M2G and PYE 9.3 mM Gln. Kan^S colonies unable to grow on M2G were screened by PCR with primers 727/728 and sequencing with primer 727.

RH1702 (NA1000 Δ*ptsH* Δ*ptsM*)

Triparental mating between RH1621 (NA1000 Δ*ptsH*), RH319 (MT607-pRK600) and RH1604 (Top10-pNPTS138-Δ*ptsM*) was selected on PYE Nal Kan, streaked on PYE Kan, cultivated o/n in PYE and plated on PYE 3% Suc. Suc^R candidates were streaked on PYE Kan and PYE. Kan^S colonies were screened by PCR with primers 814/815.

RH1727 (NA1000 Δ*ptsP* $spoT_{D81G}$)

Triparental mating between RH1758 (NA1000 Δ*ptsP*), RH319 (MT607-pRK600) and RH1743 (Top10-pNPTS138-$spoT_{D81G}$) was selected on PYE Nal Kan, cultivated o/n

in PYE and plated on PYE 3% Suc. Smallest Suc^R colonies were streaked on PYE Kan and PYE. Kan^S colonies were screened by PCR with primers 894/895 and sequencing with primer 894.

RH1728 (NA1000 Δ*spoT* $ptsP_{L83Q}$)

Triparental mating between RH1755 (NA1000 Δ*spoT*), RH319 (MT607-pRK600) and RH1737 (Top10-pNPTS138-$ptsP_{L83Q}$) was selected on PYE Nal Kan, cultivated o/n in PYE and plated on PYE 3% Suc. Smallest Suc^R colonies were streaked on PYE Kan and PYE. Kan^S colonies were screened by PCR with primers 877/878 and sequencing with primer 877.

RH1748 (NA1000 $ptsP_{L83Q}$)

Triparental mating between NA1000, RH319 (MT607-pRK600) and RH1737 (Top10-pNPTS138-$ptsP_{L83Q}$) was selected on PYE Nal Kan, cultivated o/n in PYE and plated on PYE 3% Suc. Smallest Suc^R colonies were streaked on PYE Kan and PYE. Kan^S colonies were screened by PCR with primers 877/878 and sequencing with primer 877.

RH1752 (NA1000 $spoT_{D81G}$)

Triparental mating between NA1000, RH319 (MT607-pRK600) and RH1743 (Top10-pNPTS138-$spoT_{D81G}$) was selected on PYE Nal Kan, cultivated o/n in PYE and plated on PYE 3% Suc. Smallest Suc^R colonies were streaked on PYE Kan and PYE. Kan^S colonies were screened by PCR with primers 894/895 and sequencing with primer 894.

RH1753 (NA1000 Δ*glnD* $spoT_{D81G}$)

Triparental mating between RH577 (NA1000 Δ*glnD*), RH319 (MT607-pRK600) and RH1743 (Top10-pNPTS138-$spoT_{D81G}$) was selected on PYE Nal Kan, cultivated o/n in PYE and plated on PYE 3% Suc. Smallest Suc^R colonies were streaked on PYE Kan and PYE. Kan^S colonies were screened by PCR with primers 894/895 and sequencing with primer 894.

RH1755 (NA1000 Δ*spoT*)

Triparental mating between NA1000, RH319 (MT607-pRK600) and RH1747 (Top10-pNPTS138-Δ*spoT*) was selected on PYE Nal Kan, streaked on PYE Kan, cultivated o/n in PYE and plated on PYE 3% Suc. Suc^R candidates were streaked on PYE Kan and PYE. Kan^S colonies were screened by PCR with primers 898/899.

RH1756 (NA1000 Δ*glnD* Δ*spoT*)

Triparental mating between RH577 (NA1000 Δ*glnD*), RH319 (MT607-pRK600) and RH1747 (Top10-pNPTS138-Δ*spoT*) was selected on PYE Nal Kan, streaked on PYE Kan, cultivated o/n in PYE and plated on PYE 3% Suc. Suc^R candidates were streaked on PYE Kan and PYE. Kan^S colonies were screened by PCR with primers 898/899.

RH1758 (NA1000 Δ*ptsP*)

Triparental mating between NA1000, RH319 (MT607-pRK600) and RH1757 (Top10-pNPTS138-Δ*ptsP*) was selected on PYE Nal Kan, streaked on PYE Kan, cultivated o/n in PYE and plated on PYE 3% Suc. Suc^R candidates were streaked on PYE Kan and PYE. Kan^S colonies were screened by PCR with primers 926/927.

RH1782 (NA1000 Δ*ptsM* $ptsH_{H18A}$)

Triparental mating between RH1622 (NA1000 Δ*ptsM*), RH319 (MT607-pRK600) and RH1737 (Top10-pNPTS138-$ptsH_{H18A}$) was selected on PYE Nal Kan, cultivated o/n in PYE and plated on PYE 3% Suc. Biggest Suc^R colonies were streaked on PYE Kan and PYE. Kan^S colonies were screened by PCR with primers 806/826 and sequencing with primer 826.

RH1819 (NA1000 Δ*ptsN*)

Triparental mating between NA1000, RH319 (MT607-pRK600) and RH1784 (Top10-pNPTS138-Δ*ptsN*) was selected on PYE Nal Kan, streaked on PYE Kan, cultivated o/n in PYE and plated on PYE 3% Suc. Suc^R candidates were streaked on PYE Kan and PYE. Kan^S colonies were screened by PCR with primers 963/964.

RH1829 (NA1000 Δ*ptsM* Δ*ptsN*)

Triparental mating between RH1622 (NA1000 Δ*ptsM*), RH319 (MT607-pRK600) and RH1784 (Top10-pNPTS138-Δ*ptsN*) was selected on PYE Nal Kan, streaked on PYE Kan, cultivated o/n in PYE and plated on PYE 3% Suc. Suc^R candidates were streaked on PYE Kan and PYE. Kan^S colonies were screened by PCR with primers 963/964.

RH1844 (NA1000 $spoT_{Y323A}$)

Triparental mating between RH50 (NA1000), RH319 (MT607-pRK600) and RH1817 (Top10-pNPTS138-$spoT_{Y323A}$) was selected on PYE Nal Kan, streaked on PYE Kan, cultivated o/n in PYE and plated on PYE 3% Suc. Suc^R candidates were streaked on

PYE Kan and PYE. Kan^S colonies were screened by PCR with primers 923/933 and 1175/1176.

RH1888 (NA1000 *3FLAG-ptsN*)

Triparental mating between RH50 (NA1000), RH319 (MT607-pRK600) and RH1885 (Top10-pNPTS138-*3FLAG-ptsN*) was selected on PYE Nal Kan, streaked on PYE Kan, cultivated o/n in PYE and plated on PYE 3% Suc. Suc^R candidates were streaked on PYE Kan and PYE. Kan^S colonies were screened by PCR with primers 961/964.

RH1940 (NA1000 Δ*glnD* Δ*ptsP*)

Triparental mating between RH577 (NA1000 Δ*glnD*), RH319 (MT607-pRK600) and RH1757 (Top10-pNPTS138-Δ*ptsP*) was selected on PYE Nal Kan, streaked on PYE Kan, cultivated o/n in PYE and plated on PYE 3% Suc. Suc^R candidates were streaked on PYE Kan and PYE. Kan^S colonies were screened by PCR with primers 926/927.

RH1941 (NA1000 $ptsP_{L83Q}$ Δ*glnD*)

Biparental mating between RH1748 (NA1000 $ptsP_{L83Q}$), RH528 (S17-1-pNPTS138-Δ*glnD*) was selected on PYE Nal Kan, streaked on PYE Kan, cultivated o/n in PYE and plated on PYE 3% Suc. Suc^R candidates were streaked on PYE Kan and PYE. Kan^S colonies were screened by PCR with primers 190/191.

RH1999 (NA1000 $spoT_{D81G}$ Δ*ptsN*)

Triparental mating between RH1752 (NA1000 $spoT_{D81G}$), RH319 (MT607-pRK600) and RH1784 (Top10-pNPTS138-Δ*ptsN*) was selected on PYE Nal Kan, streaked on PYE Kan, cultivated o/n in PYE and plated on PYE 3% Suc. Suc^R candidates were streaked on PYE Kan and PYE. Kan^S colonies were screened by PCR with primers 963/964.

RH2013 (NA1000 $spoT_{D81G}$ Δ*ptsH*)

Triparental mating between RH1752 (NA1000 $spoT_{D81G}$), RH319 (MT607-pRK600) and RH1605 (Top10-pNPTS138-Δ*ptsH*) was selected on PYE Nal Kan, streaked on PYE Kan, cultivated o/n in PYE and plated on PYE 3% Suc. Suc^R candidates were streaked on PYE Kan and PYE. Kan^S colonies were screened by PCR with primers 814/815.

RH2014 (NA1000 $spoT_{D81G}$ Δ*ptsN* Δ*ptsP*)

Triparental mating between RH1999 (NA1000 *spoT*$_{D81G}$ Δ*ptsN*), RH319 (MT607-pRK600) and RH1757 (Top10-pNPTS138-Δ*ptsP*) was selected on PYE Nal Kan, streaked on PYE Kan, cultivated o/n in PYE and plated on PYE 3% Suc. Suc^R candidates were streaked on PYE Kan and PYE. Kan^S colonies were screened by PCR with primers 926/927.

RH2015 (NA1000 *spoT*$_{D81G}$ Δ*ptsN* Δ*ptsH*)

Triparental mating between RH1999 (NA1000 *spoT*$_{D81G}$ Δ*ptsN*), RH319 (MT607-pRK600) and RH1605 (Top10-pNPTS138-Δ*ptsH*) was selected on PYE Nal Kan, streaked on PYE Kan, cultivated o/n in PYE and plated on PYE 3% Suc. Suc^R candidates were streaked on PYE Kan and PYE. Kan^S colonies were screened by PCR with primers 814/815.

RH2016 (NA1000 Δ*ptsP* *ptsN*$_{H66E}$)

Triparental mating between RH1758 (NA1000 Δ*ptsP*), RH319 (MT607-pRK600) and RH1523 (Top10-pNPTS138-*ptsN*$_{H66E}$) was selected on PYE Nal Kan, cultivated o/n in PYE and plated on PYE 3% Suc. Smallest Suc^R colonies were streaked on PYE Kan and PYE. Kan^S colonies were screened by PCR with primers 963/964 and sequencing with primer 1126.

RH2017 (NA1000 *ptsN*$_{H66E}$)

Biparental mating between RH50 (NA1000) and S17-1-pNPTS138-USCFP-*rodZ* [14] was selected on PYE Nal Kan. A CR30 lysate made on NA1000 pNPTS138-USCFP-*rodZ* was transduced into RH2016 (NA1000 Δ*ptsP* *ptsN*$_{H66E}$). Transductants were selected on PYE Kan, cultivated o/n in PYE and plated on PYE 3% Suc. The presence of *ptsP,* backcrossed together with USCFP-*rodZ*, was checked by PCR with primers 972/973.

RH2018 (NA1000 *ptsP*$_{L83Q}$ *ptsN*$_{H66A}$)

Triparental mating between RH1748 (NA1000 *ptsP*$_{L83Q}$), RH319 (MT607-pRK600) and RH1538 (Top10-pNPTS138-*ptsN*$_{H66A}$) was selected on PYE Nal Kan, cultivated o/n in PYE and plated on PYE 3% Suc. Biggest Suc^R colonies were streaked on PYE Kan and PYE. Kan^S colonies were screened by PCR with primers 963/964 and sequenced with primer 1126.

RH2084 (MG1655 Δ*ptsP::cat*)

cat was amplified from pKD3 by PCR using primers 1262/1263, transformed into

RH392 electrocompetent cells (pre-induced 15' at 42°C). Recombinant clones were selected on LA Cam plates at 37°C and screened by PCR with primers 1275/1276.

RH2117 (MG1655 *cyaA::frt* Δ*ptsP*)

A P1 lysate made on RH2084 (LHR106) was transduced into RH785 (MG1655 *cyaA::frt*). Transductants were selected on LA Cam. Cam^R colonies were then screened by PCR with primers 1275/1276. Candidates were then transformed with pCP20 and selected on LA Amp plates at 30°C. Clones were then streaked twice on LA plates and incubated at 42°C to get rid of *cat* cassette and pCP20. Cam^S candidates were screened by PCR with primers 1275/1276.

RH2122 (MG1655 *cyaA::frt* Δ*npr*)

A P1 lysate made on a MG1655 Δ*npr::kan* (L. Van Melderen lab) was transduced in RH785 (MG1655 *cyaA::frt).* Transductants were selected on LA Kan. Kan^R colonies were screened by PCR with primers 1310/1311 Candidates were then transformed with pCP20 and selected on LA Amp plates at 30°C. Clones were then streaked twice on LA plates and incubated at 42°C to get rid of *kan* cassette and pCP20. Kan^S candidates were screened by PCR with primers 1310/1311.

RH2124 (MG1655 *cyaA::frt* Δ*ptsP*Δ*ptsI*)

A P1 lysate made on a MG1655 Δ*ptsI::kan* (from L. Van Melderen lab) was transduced in RH2117 (MG1655 *cyaA::frt* Δ*ptsP).* Transductants were selected on LA Kan. Kan^R colonies were screened by PCR with primers 1273/1274. Candidates were then transformed with pCP20 and selected on LA Amp plates at 30°C. Clones were then streaked twice on LA plates and incubated at 42°C to get rid of *kan* cassette and pCP20. Kan^S candidates were screened by PCR with primers 1273/1274.

RH2191 (NA1000 pXMCS2-*3FLAG-ptsN*)

Triparental mating between RH50 (NA1000), RH319 (MT607-pRK600) and RH2204 (Top10-pXMCS2-*3FLAG-ptsN*) was selected on PYE Nal Kan.

RH2192 (NA1000 Δ*ptsP* pXMCS2-*3FLAG-ptsN*)

Triparental mating between RH1758 (NA1000 Δ*ptsP*), RH319 (MT607-pRK600) and RH2204 (Top10-pXMCS2-*3FLAG-ptsN*) was selected on PYE Nal Kan.

RH2193 (NA1000 $spoT_{D81G\ Y323A}$)

Triparental mating between RH1752 (NA1000 *spoT*$_{D81G}$), RH319 (MT607-pRK600) and RH1817 (Top10-pNPTS138-*spoT*$_{Y323A}$) was selected on PYE Nal Kan, streaked on PYE Kan, cultivated o/n in PYE and plated on PYE 3% Suc. Suc^R candidates were streaked on PYE Kan and PYE. Kan^S colonies were screened by PCR with primers 923/933 and 1175/1176.

RH2194 (NA1000 *ptsN*$_{H66A}$ *spoT*$_{Y323A}$)

Triparental mating between RH2019 (NA1000 *ptsN*$_{H66A}$), RH319 (MT607-pRK600) and RH1817 (Top10-pNPTS138-*spoT*$_{Y323A}$) was selected on PYE Nal Kan, streaked on PYE Kan, cultivated o/n in PYE and plated on PYE 3% Suc. Suc^R candidates were streaked on PYE Kan and PYE. Kan^S colonies were screened by PCR with primers 923/933 and 1175/1176.

RH2195 (NA1000 *ptsN*$_{H66E}$ *spoT*$_{Y323A}$)

Triparental mating between RH2017 (NA1000 *ptsN*$_{H66E}$), RH319 (MT607-pRK600) and RH1817 (Top10-pNPTS138-*spoT*$_{Y323A}$) was selected on PYE Nal Kan, streaked on PYE Kan, cultivated o/n in PYE and plated on PYE 3% Suc. Suc^R candidates were streaked on PYE Kan and PYE. Kan^S colonies were screened by PCR with primers 923/933 and 1175/1176.

RH2196 (NA1000 Δ*ptsP* *spoT*$_{Y323A}$)

Triparental mating between RH1758 (NA1000 Δ*ptsP*), RH319 (MT607-pRK600) and RH1817 (Top10-pNPTS138-*spoT*$_{Y323A}$) was selected on PYE Nal Kan, streaked on PYE Kan, cultivated o/n in PYE and plated on PYE 3% Suc. Suc^R candidates were streaked on PYE Kan and PYE. Kan^S colonies were screened by PCR with primers 923/933 and 1175/1176.

Supplementary References

1. Hogg, T., Mechold, U., Malke, H., Cashel, M. & Hilgenfeld, R. Conformational antagonism between opposing active sites in a bifunctional RelA/SpoT homolog modulates (p)ppGpp metabolism during the stringent response [corrected]. *Cell* **117,** 57–68 (2004).
2. Thanbichler, M., Iniesta, A. A. & Shapiro, L. A comprehensive set of plasmids for vanillate- and xylose-inducible gene expression in Caulobacter crescentus. *Nucleic Acids Research* **35,** e137–e137 (2007).
3. Schofield, W. B., Lim, H. C. & Jacobs-Wagner, C. Cell cycle coordination and regulation of bacterial chromosome segregation dynamics by polarly localized proteins. *The EMBO Journal* **29,** 3068–3081 (2010).
4. Boutte, C. C. & Crosson, S. The complex logic of stringent response regulation in Caulobacter crescentus: starvation signalling in an oligotrophic environment. *Molecular Microbiology* **80,** 695–714 (2011).
5. Karimova, G., Pidoux, J., Ullmann, A. & Ladant, D. A bacterial two-hybrid system based on a reconstituted signal transduction pathway. *Proc. Natl. Acad. Sci. U.S.A.* **95,** 5752–5756 (1998).
6. Cherepanov. Gene disruption in Escherichia coli. *Gene* 1–6 (2003).
7. Guzman, L. M., Belin, D., Carson, M. J. & Beckwith, J. Tight regulation, modulation, and high-level expression by vectors containing the arabinose PBAD promoter. *Journal of Bacteriology* **177,** 4121–4130 (1995).
8. Gonzalez, D. & Collier, J. Effects of (p)ppGpp on the Progression of the Cell Cycle of Caulobacter crescentus. *Journal of Bacteriology* **196,** 2514–2525 (2014).
9. Simon, R., Priefer, U. & Pühler, A. A broad host range mobilization system in vivo genetic engineering: transposon mutagenesis in gram negative bacteria. **1,** 784–791 (1983).
10. Casadaban, M. J. & Cohen, S. N. Analysis of gene control signals by DNA fusion and cloning in Escherichia coli. *J. Mol. Biol.* **138,** 179–207 (1980).
11. Abel, S. *et al.* Regulatory cohesion of cell cycle and cell differentiation through interlinked phosphorylation and second messenger networks. *Molecular Cell* **43,** 550–560 (2011).
12. Evinger, M. & Agabian, N. Envelope-associated nucleoid from Caulobacter crescentus stalked and swarmer cells. **132,** 294–301 (1977).
13. Hughes, H. V. *et al.* Protein localization and dynamics within a bacterial organelle. *Proceedings of the National Academy of Sciences* **107,** 5599–5604 (2010).
14. Alyahya, S. A. *et al.* RodZ, a component of the bacterial core morphogenic apparatus. *Proceedings of the National Academy of Sciences* **106,** 1239–1244 (2009).
